# Ultra‐Low Hysteresis Under Large Deformation Enabled by Fast Chains Relaxation in Highly Competitive Dynamic Hydrogen Bond Networks

**DOI:** 10.1002/advs.202505417

**Published:** 2025-06-25

**Authors:** Shuaijun Guo, Shilei Zhu, Yang Qiao, Shanhao Feng, Xin Yang, Beibei Kang, Chaojun Yue, Yanjing Zhang, Zhuangzhuang Li, Ya Nan Ye, Qiang Zheng

**Affiliations:** ^1^ College of Materials Science & Engineering Taiyuan University of Technology Taiyuan 030024 China; ^2^ Shaanxi Key Laboratory of Chemical Additives for Industry Xi'an Key Laboratory of Advanced Performance Materials and Polymers Shaanxi University of Science and Technology Xi'an 710021 China; ^3^ Department of Polymer Science and Engineering Zhejiang University Hangzhou 310027 China

**Keywords:** deep eutectic solvent, dynamic bonds, hydrogen bonds, ultrafast response, ultra‐low hysteresis

## Abstract

High‐quality capture of diverse motion signals in flexible sensors requires soft sensing materials to perform signal conversion and transmission stably and without delay over extended periods. However, low hysteresis achieved through purely elastic mechanisms easily exhibits poor crack propagation resistance. By leveraging the significant gap between the strain rate during large‐strain service conditions and the crack propagation rate in the fracture process, this study presents a facile strategy for constructing a highly competitive dynamic hydrogen bonding system to produce near‐zero‐hysteresis and highly crack‐resistant D‐gels. Through tuning the relaxation dynamics of strong hydrogen bonding interactions with polymer segments by insert deep eutectic solvent (DES) components, the highly dynamic hydrogen bonds are rendered mechanically “invisible” during service condition—an essential factor in achieving a low‐hysteresis attribute (low hysteresis: <3%). Meanwhile, the relaxation time of those dynamic bonds is comparable to the inverse of crack propagation rate, effectively alleviating stress concentration at the crack tips, thereby enhancing the ultimate fracture strain (1500%) and crack propagation strain (550%) of the gels. This approach provides a general strategy for synthesizing gels that overcome the traditional trade‐off between high crack propagation resistance and high elasticity.

## Graphical abstract

This study demonstrates that incorporating deep eutectic solvent (DES) components tunes the relaxation dynamics of strong hydrogen bonds between polymer segments, rendering them mechanically “invisible” during service conditions (low hysteresis: <3%). These dynamic bonds alleviate stress concentration at crack tips, significantly enhancing the gels’ ultimate fracture strain (1500%) and crack propagation strain (550%).

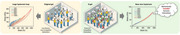

## 1 Introduction

Large volumes of high‐quality motion data collection for high‐throughput data processing by flexible sensors urgently requires for efficient and accurate information sensing and simplified material fabrication techniques to meet the demands of large‐scale applications and industrialization.^[^ ^1^, ^2^, ^3^, ^4^, ^5^, ^6^ ^]^ Hydrogels, composed of a 3D network of little amount of hydrophilic polymer chains that can absorb large amounts of water, exhibits pure elastic and low‐hysteresis due to negligible friction between sparse polymer chains, which make hydrogels ideal materials for flexible sensor^[^ ^7^, ^8^, ^9^, ^10^, ^11^, ^12^ ^]^ that respond to external stimuli such as light,^[^ ^13^ ^]^ temperature,^[^ ^14^ ^]^ magnetic fields,^[^ ^15^ ^]^ and pressure,^[^ ^16^, ^17^ ^]^ demonstrating significant potential in applications including wearable electronics,^[^ ^13^, ^15^ ^]^ soft electronics,^[^ ^14^, ^15^ ^]^ and health monitoring.^[^ ^15^, ^16^, ^17^, ^18^ ^]^ However, the conventional hydrogels always are inherently heterogeneous due to the large distribution of length of network strands between crosslinkers. Under deformation, the shortest network strand reaches its full chain extensibility at very small macroscopic strain and ruptures, causing the stress it carried to redistribute among the neighboring intact strands. This redistribution increases the force loaded on these neighboring strands, leading to further damage of the neighboring strands, eventually triggering an avalanche rupture and catastrophic failure of the sample, rendering the gels suffer from low mechanical strength and poor toughness, making them unsuitable for repeated use under varying mechanical loads and deformation conditions.^[^ ^19^ ^]^ Over the past two decades, strategies such as double networks,^[^ ^20^ ^]^ freeze‐thaw cycles,^[^ ^21^ ^]^ and the Hofmeister effect^[^ ^22^ ^]^ have been employed to toughen hydrogels, making their toughness comparable to that of human cartilage.^[^ ^23^ ^]^ However, these toughening strategies, which rely on the introduction of sacrificial bonds or reversible bonds as energy‐dissipating structures,^[^ ^24^, ^25^ ^]^ often result in significant mechanical hysteresis (ranging from 20% to 60%) at the macroscopic level due to the energy dissipation and the delayed recovery of the polymer network after deformation.^[^ ^26^, ^27^ ^]^ This delayed recovery can further disrupt the pathways for electron transport within the material, thereby reducing the efficiency of electron conduction and, ultimately, the sensitivity of sensors made from such hydrogels.^[^ ^28^, ^29^ ^]^ To reduce the mechanical hysteresis of tough hydrogels, it is essential to enhance the mobility of polymer chains, namely, to quicken chain relaxation dynamics to enabling network recovery.^[^ ^30^, ^31^, ^32^, ^33^ ^]^ Taking the advantage of fast relaxation nature of entanglements, Suo et al. fabricated the single‐network hydrogels with highly entangled structures, in which the extensive chain entanglement serves as a slippage point, allowing stress to be distributed across other chains with minimal friction, thereby reducing hysteresis to a negligible level.^[^ ^34^ ^]^ However, this low‐hysteresis behavior is typically achieved by constructing purely elastic polymer networks, which often exhibit poor crack propagation resistance. As a result, these systems are highly sensitive to defects in practical applications, leading to constraints in their lifespan and applicability, particularly during large‐scale and intense sensor data collections. In some cases, the inherent mechanical limitations of the material result in low elongation, preventing the achievement of large deformations and thereby failing to attain low hysteresis under such conditions.^[^ ^34^, ^35^ ^]^

Low mechanical hysteresis can also be achieved through sophisticated molecular structure design. Ito et al. developed a slide‐ring (SR) hydrogel using a spatially constrained gelation strategy.^[^ ^36^ ^]^ These SR‐designed hydrogels exhibit minimal hysteresis due to strain‐induced crystallization of the oriented polyethylene glycol (PEG) chains, enabling efficient energy dissipation and nearly complete recovery without irreversible damage. Yan et al. minimized chain segment slippage in hydrogels through nanoconfined polymerization of porous cationic polymers with adjustable counterions, achieving excellent hysteresis properties even under large deformations.^[^ ^37^, ^38^ ^]^ However, hydrogels created through such precise molecular design often involve high costs and complex manufacturing processes, limiting their scalability and widespread application.

Herein, we propose a timescale‐selective mechanical response masking strategy for producing near‐zero‐hysteresis and highly crack‐resistant D‐gels capable of withstanding a wide range of deformations by constructing a highly competitive dynamic hydrogen bonding system through the introduction of deep eutectic solvent (DES) components. DES have tunable hydrogen bond strength and enhanced molecular mobility due to the diversity of hydrogen bond donors (HBDs) and acceptors (HBAs).^[^ ^39^ ^]^ These properties, combined with their strong hydrogen‐bonding energies, enable them to effectively break and form hydrogen bonds, allowing for the dynamic reconstruction of the bonding network in hydrogels.^[^ ^40^, ^41^ ^]^ Tough D‐gels are prepared through facile one‐step radical polymerization incorporating specifically designed highly competitive dynamic hydrogen bonding system. The relaxation dynamics of hydrogen bonding interactions with polymer segments are tuned by inserting DES components, rendering hydrogen bonds mechanically “invisible,” which is crucial for achieving a low‐hysteresis attribute (low hysteresis: 1.57%) during the loading and unloading of forces under large deformation conditions (1500%). When the relaxation time of the hydrogel bonds is longer than the crack propagation time, it alleviates stress concentration at the crack tips and prevents crack propagation, thereby enhancing the ultimate fracture strain (1500%) and crack propagation strain (550%) of the hydrogel. This approach provides a general strategy for synthesizing gels, overcoming the traditional trade‐off between high crack propagation resistance and high elasticity. Additionally, D‐gels exhibit ultra‐stretchability (1500%), fast response (102 ms), and good water retention and antifreeze performance. These unique features, along with the simple preparation process, make this low‐hysteresis hydrogel highly promising for flexible sensors and offer a valuable approach for advancing the large‐scale application and industrial development of smart devices.

## 2 Results and Discussion

### 2.1 Designing A Highly Competitive Hydrogen Bonding System for Ultra‐Low‐Hysteresis Featured D‐Gels

**Figure 1:**
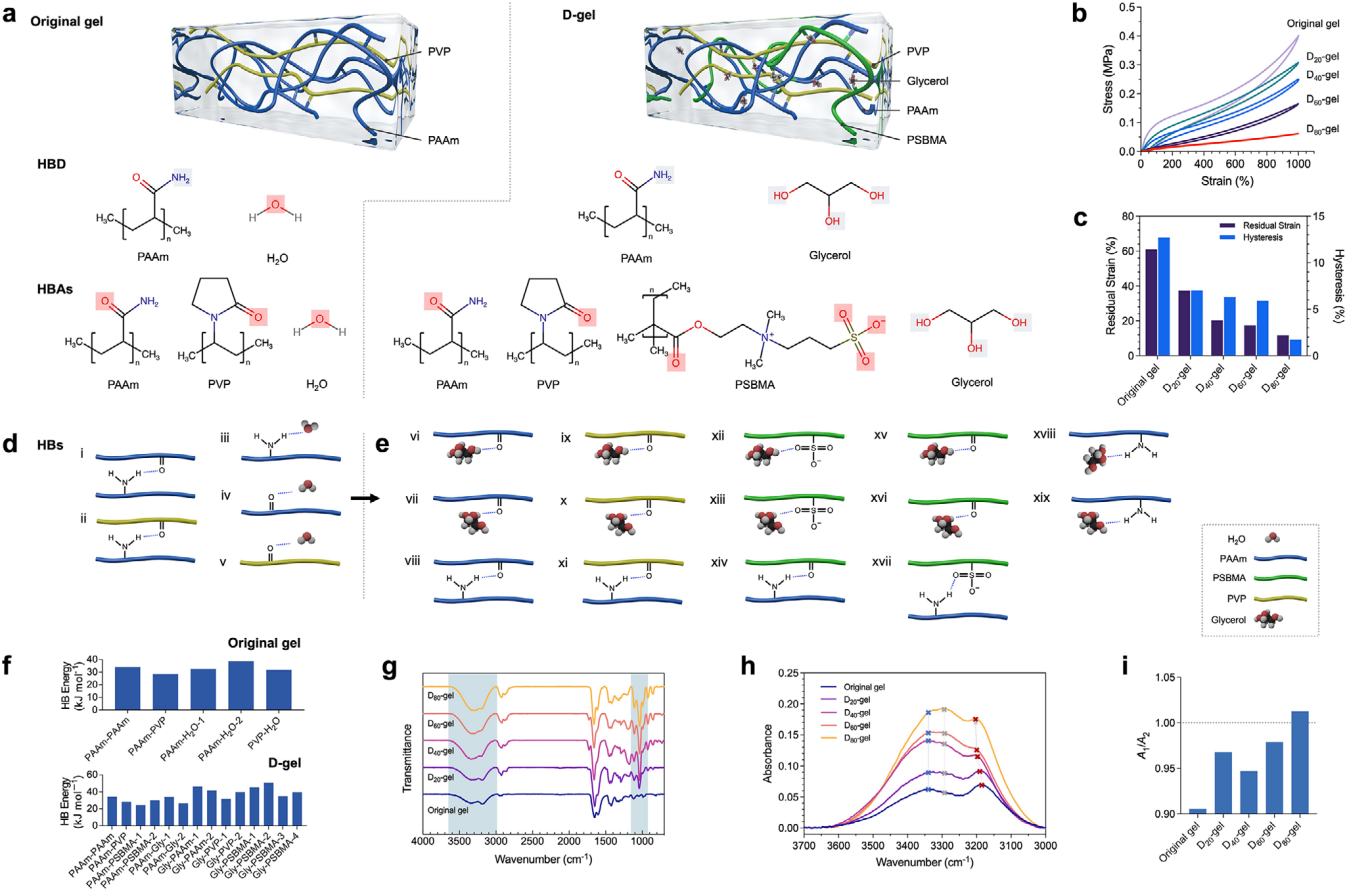
Design and low‐hysteresis characteristics of highly dynamic hydrogen bonding D‐gels. a) Schematic illustration of the polymer network structure with their respective hydrogen bond donors (HBDs) and hydrogen bond acceptors (HBAs) of the original gel, composed of PAAm (blue) and PVP (yellow) polymers, and D‐gels, which incorporates a more complex combination of PAAm (blue), poly(sulfobetain methacrylate) (PSBMA, green), PVP (yellow), and glycerol. b) Stress–strain curves showing the mechanical performance of the original gels and D‐gels under cyclic loading c) with corresponding value comparison of residual strains and hysteresis loss. d) Illustration of the potential hydrogen bonding interactions among PAAm, PSBMA, PVP, and glycerol molecules, highlighting the formation of a competitive bonding environment in D‐gels. e) Bar graphs showing the hydrogen bond energy under various possible configurations, demonstrating the enhanced diversity of dynamic hydrogen bonding in D‐gels. f) The hydrogen bond energies for all potential hydrogen bond interactions within the original gel and the D_80_‐gel were evaluated. g) The FTIR spectra of both the D‐gels and the original gel were recorded, highlighting the O‐H stretching band and the N‐H stretching band, which are indicated by shaded areas in the spectra. h) The infrared absorption spectra were analyzed over a wavelength range of 3000 to 3700 cm^−1^ to assess the molecular vibrational characteristics. i) The FTIR absorbance ratios (A_1_ and A_2_) were calculated at two specific wavenumbers, 3283 and 3331 cm^−1^, to provide insights into the relative intensities of the corresponding functional groups in the gels.

Our approach is based on a dual‐network structured original gel consisting of acrylamide (AAm) monomers with hydrophilic and amide groups, and polyvinylpyrrolidone (PVP) with high molecular flexibility and hydrophilic properties. The AAm monomers can form covalent bonds through radical polymerization, while the PVP polymer chains interact with the polyacrylamide (PAAm) network through dynamic hydrogen bonds, creating a stable and extensive hydrogen bonding mechanism within the double‐network structure, as illustrated in **Figure** **1**a,d. Due to the imbalance and inconsistent reformation of these dynamic hydrogen bonds under stress, this system typically exhibits significant hysteresis effects (Figure 1b).

To ensure no hysteresis in a dynamic bond gel system, the breaking and reformation of the dynamic bonds must occur very rapidly and near synchronously, allowing the bonds to immediately return to their equilibrium state after the application and release of stress.^[^ ^1^, ^42^ ^]^ We introduced a DES component, as a hydrogen bonding competitor containing sulfobetain methacrylate (SBMA) and glycerol (Gly), into the original gel system to reconstruct the dynamic bonding network configuration as illustrated in Figure 1a. A series of thermal analysis data of the SBMA‐ glycerol mixtures confirmed the formation of SBMA/ Gly DESs (as shown in Figures S1 and S2, Supporting Information). SBMA, as a monomer, is cross‐linked into the hydrogel network. According to the “hydrogen bond continuous association” theory,^[^ ^43^, ^44^, ^45^ ^]^ the carbonyl and sulfonate groups (−SO_3_^−^) on SBMA act as multiple hydrogen bond acceptors (HBAs), creating competitive hydrogen bonding sites that interact with the original network and other components within the system. Additionally, glycerol, with its multiple hydroxyl groups acting as hydrogen bond donors (HBDs), interacts with the polymer networks by forming hydrogen bonds, thereby increasing the diversity of hydrogen bonding interactions, further improving the gel's flexibility and dynamic response.^[^ ^46^, ^47^ ^]^ Our introduction of SBMA‐Gly DES increases the variety of HBDs and HBAs in the system (Figure 1a), greatly enhancing the diversity of potential hydrogen bond combinations as shown in Figure 1e.

We refer to the gel samples with DES components incorporated as “D‐gels”. More specifically, when discussing the DES content, we denote them as “D*_x_*‐gels”, where “*x*” represents the mass percentage of SBMA and glycerol in the DES used. The DES content is kept constant across all samples. Figure 1d shows the uniaxial tensile stress–strain curves of a D_80_‐gel sample in comparison with the original gel reference at a fixed strain of 1000%. Compared to the large hysteresis loop displayed by the original gel reference, the D_80_‐gel sample shows nearly overlapping stress‐ = strain curves during loading and unloading, demonstrating the rapid mechanical response in this gel system. Accordingly, as shown in Figure 1e, the residual strain and hysteresis rate of the hydrogel samples before and after DES incorporation were compared. For the D_80_‐gel sample, the residual strain significantly decreased from 60.95% to 11.8%, and the hysteresis rate dropped substantially from 12.1% to 1.75% compared to the original gel. Replacement of DES with glycerol alone resulted in significantly greater hysteresis loss (15%) and residual strain (51%), underscoring the crucial role of DES in facilitating such low hysteresis in D_80_‐gel (Figure S3, Supporting Information.).

The introduction of DES significantly enhances the diversity of hydrogen bonds and the competition among HBDs and HBAs within the system, which is proposed to be a key factor in achieving the near‐zero hysteresis feature in D‐gels.^[^ ^48^, ^49^ ^]^ Through density functional theory (DFT) calculations, we evaluated the hydrogen bond energies of all potential hydrogen bonding interactions in the gel (Figure 1f)(Figures S4 and S5, Supporting Information). The calculation results show that the newly introduced hydrogen bonds demonstrate more pronounced strength and higher formation probability compared to the structural network hydrogen bonds in the original gel, while maintaining the overall uniformity of hydrogen bond strength within the system. Specifically, the new hydrogen bonds formed between glycerol and SBMA, glycerol and PVP, as well as glycerol and AAm are not only stronger but also easier to form, thanks to the diverse and crowded environment of introduced non‐aqueous HBDs. In the original gel, the energy dissipation mechanism arises from robust hydrogen bonding interactions between polymer chains.^[^ ^50^, ^51^ ^]^ Upon introducing DES components, these interactions are weakened due to the formation of several new, strong, and flexible hydrogen bond combinations (Gly‐AAm, Gly‐PSBMA, Gly‐PVP). These new interactions create a more crowded atmosphere that makes the breaking and reformation of hydrogen bonds between chains more randomized, further enhancing the ease and flexibility of switching inter‐chain hydrogen bonding configurations within the system.

We used FTIR to further explore the hydrogen bonding characteristics in D‐gels. The FTIR spectra of D‐gels and the original gel show that the introduction of DES into the gel system significantly alters the hydrogen bonding environment (Figure 1g). Specifically, at the range of 3000 to 3700 cm^−1^, the absorbance increases with more DES component incorporated, indicating a higher density of hydrogen bonds in the system. This is consistent with the idea that introducing DES increases the number of HBDs (like hydroxyl groups from glycerol) and HBAs (like sulfonate groups from SBMA), leading to a much denser hydrogen bond network. It is also worth noting that we observed a slight blue shift in the N‐H stretching band as the DES content increased (Figure 1h). Typically, hydrogen bonding causes a redshift by weakening the bond strength of the functional groups involved, resulting in lower vibrational frequencies.^[^ ^52^, ^53^ ^]^ This blue shift suggests that, after the introduction of DES, the role of polyacrylamide's N‐H groups in forming hydrogen bonds is shared with a more diverse and competitive hydrogen bonding network, compared to the simpler, polyacrylamide‐dominated hydrogen bond network in the original gel. Due to the broad nature of the O‐H stretching vibration band, which reflects the diversity of hydrogen bonding environments, we compared the FTIR absorbance ratios (*A*_1_ and *A*_2_) at two fixed wavenumbers, 3283 and 3331 cm^−1^, for each sample to analyze the shift in the O‐H band. We found that this ratio (*A*_1_/*A*_2_) increases with the introduction of DES, indicating a redshift in the O‐H stretching band. This suggests a progressive strengthening of the hydrogen bonds contributed by hydroxyl groups across the system.

**Figure 2:**
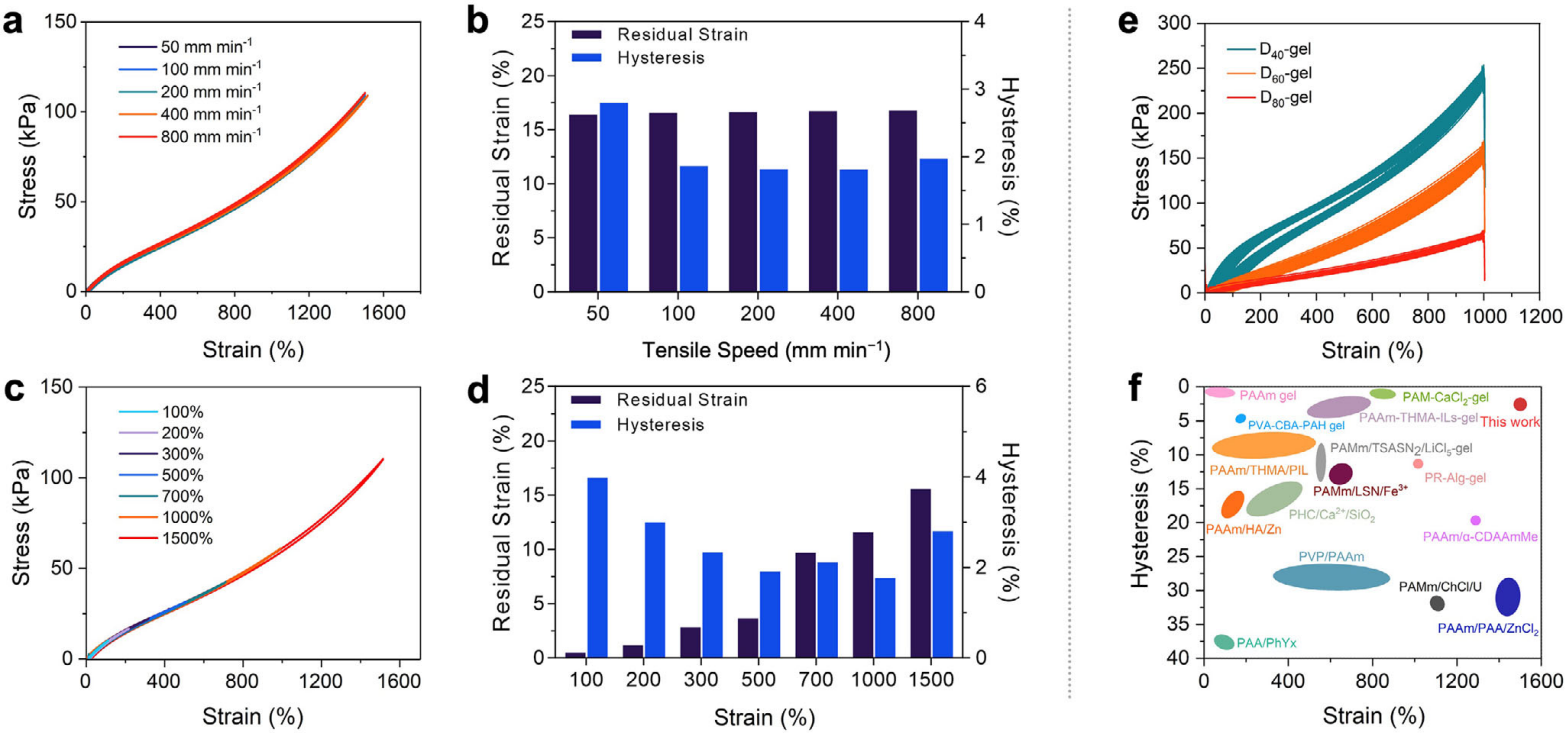
Mechanical performance and low‐hysteresis features of D_80_‐gel. a,c) Stress–strain curves d) with corresponding residual strain and hysteresis rate of D_80_‐gel under increasing cyclic strains (100–1500%) at a tensile speed of 100 mm min^−1^, demonstrating a well‐maintained structure and minimal hysteresis, even under large deformations. e) The stress–strain curves for the D‐gels under 200 consecutive tensile cycles (D_40_, D_60_, and D_80_‐gels). f) Comparison of hysteresis (%) vs. strain (%) for D‐gels and other previously reported systems, with D_80_‐gel in this work exhibiting significantly lower hysteresis across a wide range of strains.

To further investigate the hysteresis performance of the representative D_80_‐gel, we conducted a series of tensile tests under various conditions. The digital photograph of the mechanical properties of D_80_‐gel is shown in Figure S6 (Supporting Information). **Figure** **2**a shows the uniaxial tensile stress–strain curves at a fixed strain of 1500% under various stretching rates. We observed that across the selected range of stretching rates (50–800 mm min^−1^), the stress–strain curves during the loading‐unloading cycles were nearly identical, indicating minimal hysteresis even under large deformation, with the hysteresis rate consistently staying below 3% (Figure 2b). Importantly, as the stretching rate increased from 50 to 800 mm min^−1^, the changes in residual strain and hysteresis rate during each cycle were almost negligible. This demonstrates that the hysteresis characteristics of the D_80_‐gel are largely unaffected by stretching rates across an order of magnitude (Figure 2b). (The stress–strain curves for the remaining gels (original, D_20_, D_40_, D_60_‐gel) at different stretching speeds are shown in Figure S7, Supporting Information.)

To demonstrate the low hysteresis characteristics of the gel under varying deformation scenarios, we conducted tensile loading‐unloading tests on the D_80_‐gel at a fixed stretching rate of 100 mm min^−1^, with gradually increasing strain ranges from 100% to 1500%. As shown in Figure 2c, during multiple stretching tests, all the stress–strain curves for stretching and relaxation nearly overlapped. As the strain increased 15‐fold, the stress rose from 16 to 113 kPa, the residual strain increased from 0.23% to 16%, and the energy dissipated calculated from the strain‐stress curve increased from 0.08 kJ m^−3^ to a still relatively low level of 2.54 kJ m^−3^. Accordingly, the hysteresis rates of the hydrogel were calculated for each test, with the D_80_‐gel maintaining a hysteresis rate below 5% (Figure 2d; Figure S8, Supporting Information). This demonstrates that the low hysteresis properties of D_80_‐gel are unaffected over a wide range of tensile strains. (The stress–strain curves for the remaining gels (original, D_20_, D_40_, D_60_‐gel) under increasing strain at a fixed speed of 100 mm min^−1^ are presented in Figure S9, Supporting Information.)

Notably, under large deformations (1500% strain), the gel exhibits slightly smaller hysteresis compared to small deformations (100% strain). According to the entropic elasticity theory for polymers, at low strains, polymer chains are still adjusting, rearranging, and stretching, which leads to greater energy dissipation accompanied with a significant decrease in the entropy of the polymer network.^[^ ^35^, ^54^ ^]^ However, once the chains are fully extended, further deformation does not cause as much decrease in entropy, resulting in less energy dissipation and reduced hysteresis.^[^ ^55^, ^56^, ^57^ ^]^ In addition, We also tested the D_80_‐gel under multiple tensile cycles at different strain gradients (see Figure S10, Supporting Information), where each set of cycles exhibited highly similar curves, further confirming its high mechanical stability and reliability.

Figure 2e shows the stress–strain curves for the hydrogel under 200 consecutive tensile cycles at a fixed strain of 1000% and a stretching rate of 400 mm min^−1^. Except for the first cycle, where the hysteresis loop is slightly larger due to the breaking of strong interfacial chemical bonds between polymer chains, the loading‐unloading curves for the remaining cycles are highly overlapping. This indicates the formation of a recoverable and stable network during the cycling process, demonstrating the gel's excellent fatigue resistance and reliability even under large deformation. (Cycling data for three sets of gels (D_80_, D_60_, and D_40_‐gels) are shown here, and cycling curves for D_20_ and the original gel are shown in Figure S11, Supporting Information) Compared to related works previously reported, our D‐gels exhibit superior performance in terms of hysteresis behavior under extensive deformations (Figure 2f). (Table S2, Supporting Information)

Compared to conventional ChCl‐based DESs, such as ChCl/Gly, which provide limited yet measurable hydrogen bonding interactions, the SBMA/Gly DES establishes a more competitive and diverse hydrogen bonding network due to the presence of multiple donor and acceptor groups. To evaluate this difference, we synthesized control hydrogels (C*_x_*‐gels, *x* denotes the weight percentage of DES) using a 3:1 Gly:ChCl DES, and found that their hysteresis behavior was consistently higher than that of D_80_‐gel. At 1000% strain, C_80_‐gel exhibited a hysteresis rate of 7.9% and a residual strain of 53%, confirming that the SBMA/Gly DES offers superior resistance to energy dissipation under large deformation (Figure S12, Supporting Information).

### 2.2 Crack Propagation Resistance

Typically, a larger hysteresis loop indicates significant energy dissipation due to the presence of dynamic sacrificial bonds during the loading and unloading cycles. In both the original gel and the D‐gels systems we discussed above, there are numerous hydrogen bonds that can act as sacrificial bonds for energy dissipation. However, as shown in Figure 1d, the disappearance or “flattening” of the hysteresis loop in the D‐gels raises the question: does this mean that the hydrogen bonds in this system lack energy dissipation characteristics? Alternatively, could it suggest that the highly competitive hydrogen bonding network in the D‐gels system accelerates the breaking and reformation of bonds during loading and unloading, resulting in a rapid response that exceeds the resolution of the mechanical testing system and leads to a greatly reduced hysteresis effect on a macroscopic scale?

Good crack propagation resistance is essential for the long‐term operational stability of flexible sensor materials under large deformations. Building on these considerations and to address the above questions regarding the dynamic hydrogen bond behavior and hysteresis of the D‐gels system, we investigated its crack propagation characteristics and found that the D‐gels exhibits excellent resistance to crack propagation. To demonstrate the presence of fast hydrogen bond breaking and reformation, which act as short‐lived energy dissipation structures in the D‐gels, we compared its crack propagation characteristics to those of pure PAAm hydrogels, which lack dynamic dissipation structures. As an extreme example of a strategy that continually shortens dynamic bond lifetimes, PAAm hydrogels exhibit purely elastic behavior with minimal internal friction and energy dissipation when the polymer chains are stretched. Consequently, they exhibit almost no mechanical hysteresis during loading and unloading cycles (Figure S13, Supporting Information).^[^ ^58^, ^59^, ^60^ ^]^ However, this type of single‐network hydrogel with purely entropic elasticity has poor mechanical properties and low toughness, making it unsuitable for use under large strains.

**Figure 3:**
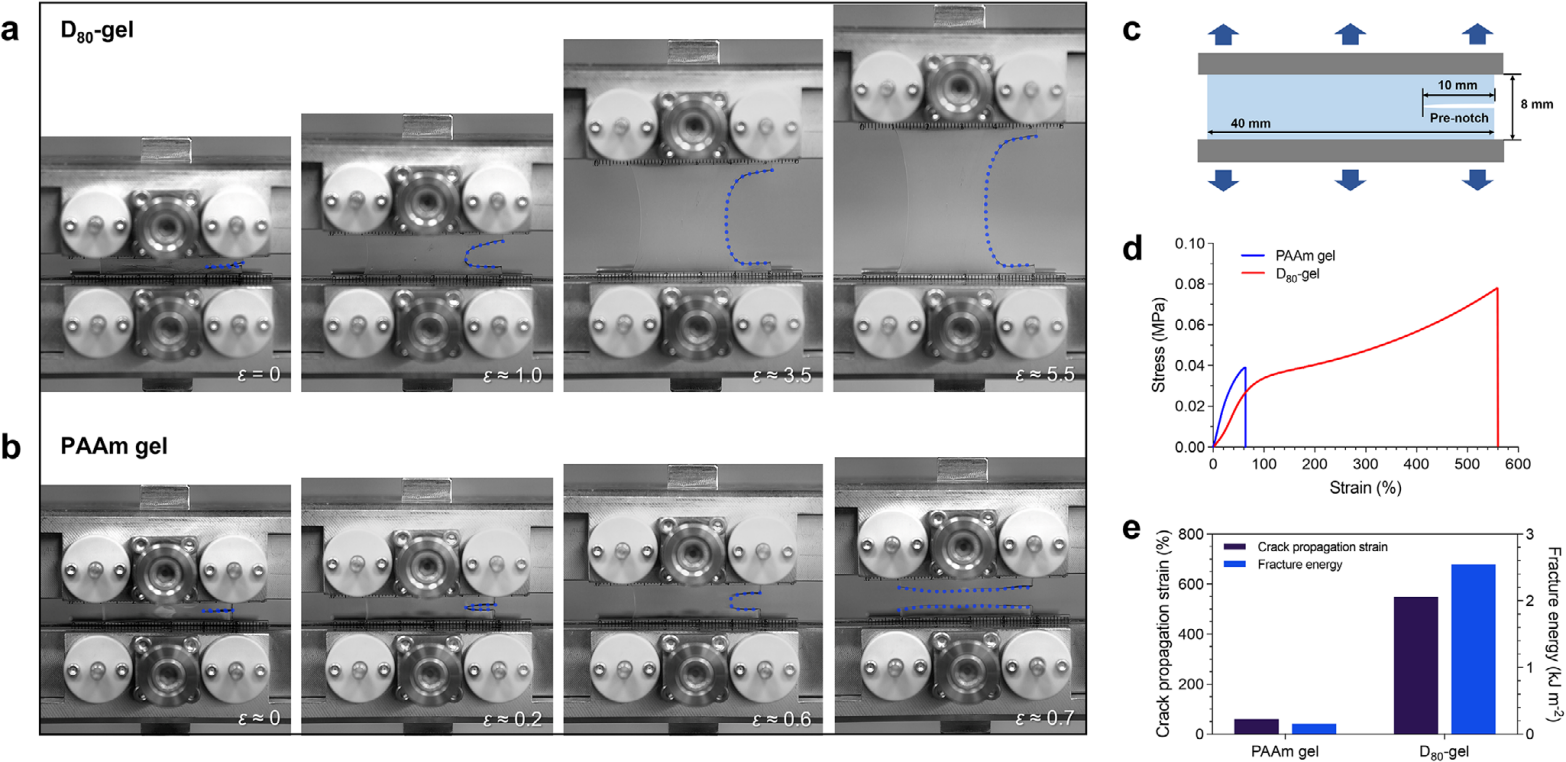
Crack propagation behavior comparison between D_80_‐gel and PAAm hydrogel. a) The crack blunting observed at *ε* ≈ 1.0 and gradual crack propagation up to *ε* ≈ 5.5 of a notched D_80_‐gel sample indicates the its resistance to crack growth. b) The rapid crack propagation in a notched PAAm hydrogel sample at a low strain. c) Schematic diagram of crack extension gel specimen. d) Stress–strain curves for notched PAAm and D_80_‐gel hydrogel samples under tensile testing. e) Quantitative comparison of crack propagation strain and fracture energy between PAAm and D_80_‐gel.

To evaluate the crack propagation sensitivity of PAAm hydrogels and D‐gels, we used standardized single‐edge notched specimens to test the ultimate fracture strain under pure shear loading. **Figure** **3**a shows that due to the lack of an energy dissipation mechanism, the PAAM hydrogel experiences stress concentration at the crack tip, leading to rapid crack propagation at strains around *ε* ≈ 0.6. Unlike PAAm, the D_80_‐gel shows a much more gradual crack progression even under higher strains, starting from *ε* ≈ 1 and extending to *ε* ≈ 5.5 (Figure 3b). This difference is further illustrated in Figure 3c, where the stress–strain curve of the PAAm hydrogel shows a sharp increase in stress followed by a sudden drop, indicating quick crack propagation at a strain of 0.6. While the D_80_‐gel exhibits a gradual stress increase and a broader curve, with crack blunting occurring up to a strain of 2.0. We found that D‐gels in a wide range of compositions consistently demonstrate similarly excellent crack propagation resistance, as shown in Figure S14 (Supporting Information). Figure 3d quantifies these observations, showing that the crack propagation strain for the D_80_‐gel is ≈550%, and its fracture energy reaches 2.54 kJ m^−2^, which are ≈9 times and 16 times higher than those of the PAAm hydrogel, respectively. The exceptional crack resistance of the D‐gels suggests the presence of energy dissipation structures based on dynamic hydrogen bond breaking and reformation within the gel system during deformation.

Building on our earlier observation of the extremely low hysteresis in D‐gels, we hypothesize that the highly competitive hydrogen bond network in the D‐gels system significantly enhances the dynamic behavior of the hydrogen bonds, leading to rapid breaking and reformation. This fast reconstruction of the hydrogen bond network during deformation contributes to energy dissipation, thereby improving the crack propagation resistance of the D‐gels. However, due to the limited “resolution” of the mechanical testing equipment, these rapid dynamic events are not captured, resulting in an almost low‐hysteresis behavior on a macroscopic scale. This also suggests that the dynamic bond lifetimes in the D‐gels system are much shorter than the observation window during testing, rendering the dynamic dissipation structures mechanically “invisible”.

### 2.3 Rheological Analysis of D‐Gels

**Figure 4:**
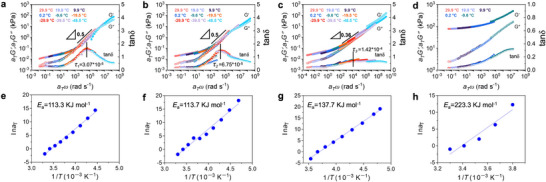
Rheological analysis of D‐gels on hydrogen bond dynamics. Time‐temperature superposition (TTS) master curves for a) D_80_‐gel, b) D_60_‐gel, c) D_40_‐gel and d) the original gel reference at various temperatures, showing the frequency‐dependent mechanical behavior under low‐temperature rheological conditions. Arrhenius plots for d) D_80_‐gel, e) D_60_‐gel, f) D_40_‐gel, and g) the original gel reference used to calculate the activation energies, showing a continuous decrease from 223.2 kJ mol^−1^ for the original gel to 113.4 kJ mol^−1^ for the D_80_‐gel. The slopes of 0.5 in (a) and (b), marked with right‐angled triangles, indicate Rouse model behavior, further demonstrating the enhanced flexibility of molecular chains in the D‐gels due to the effect of the DES components.

The breaking and reformation of hydrogen bonds in the gel system are essentially viscoelastic relaxation processes. If the breaking and reformation occur at slower rates, the relaxation process becomes more pronounced, leading to greater irreversible dissipation of viscoelastic energy. To explore the impact of DES on the dynamic behavior of hydrogen bonds in D‐gels, we employed the time‐temperature superposition (TTS) principle using rheological shift factors *a*_T_ to construct the TTS master curves for different hydrogels. The initial storage modulus, loss modulus, and loss factor of D‐gels are shown in Figure S15 (Supporting Information). **Figure** **4**a–d presents the master curves for D_80_‐gel, D_60_‐gel, D_40_‐gel, and the original gel at various temperatures, revealing their frequency‐dependent mechanical behavior under low‐temperature rheological conditions. The relaxation time (*τ*) of each gel was determined from the shifted frequencies, and the corresponding activation energy was calculated using the Arrhenius equation, *k* = *Ae* ^−^ *^E^* ^a/^ *^RT^*, where *k* is the rate constant, *A* is the pre‐exponential factor, *E*_a_ is the activation energy, *R* is the universal gas constant, and *T* is the temperature.

With the introduction of DES content into the system, both the relaxation time and activation energy decrease, and the extent of this decrease intensifies as the DES content increases. Specifically, D_80_‐gel exhibits a relaxation time of 3.07 × 10^−5^ s and an activation energy of 113.4 kJ mol^−1^ (Figure 4d), while D_40_‐gel shows a longer relaxation time of 1.42 × 10^−4^ s and a higher activation energy of 137.7 kJ mol^−1^ (Figure 4f). Compared to the D‐gels, the original gel exhibits a longer relaxation time and higher activation energy, indicating much lower network dynamics and chain mobility in the original gel. The lower activation energy and shorter relaxation time in D‐gels suggest that the introduction of highly competitive hydrogen bonding sites facilitates easier chain movement with much reduced the energy barrier.

These activation energy results demonstrated a weakening of overall hydrogen bond strength accompanied by a dramatic increase in hydrogen bonding density. This balance of weaker, more dynamic bonds and higher bonding density enables rapid network rearrangement, which underpins the enhanced mechanical adaptability and reduced hysteresis observed in D‐gels.

The slopes of 0.5 are observed in Figure 4a,b, which suggests that the polymer chains in the D‐gels system follow the Rouse model, meaning that the molecular dynamics are primarily governed by local segmental motion of the polymer chains, rather than large‐scale interactions like chain entanglements. This type of motion is typical of polymers in a random coil state, where the chains are flexible and their segments can move relatively freely, indicating that the introduction of DES enhances this flexibility by allowing easier motion of the chains within the system, even as the hydrogen bond density significantly increases.

To further investigate hydrogen bond dynamics, we conducted molecular dynamics (MD) simulations. Compared to the original gel, the D_80_‐gel exhibits a significant increase in both the variety and quantity of inter‐chain hydrogen bonds (Figure S16, Supporting Information), greatly enhancing the spatial competitiveness of these interactions. Combined with density functional theory (DFT) calculations, the energy of hydrogen bonds formed between the main chains of the D_80_‐gel is found to be comparable (Figure 1f). This combination of findings confirms the intense competition between the instantaneous formation and breaking of hydrogen bonds within the system. Additionally, analysis of the hydrogen bond correlation function *C*(*t*) (Figure S16, Supporting Information) reveals distinct dynamic behavior between the original gel and the D_80_‐gel. The original gel displays a relatively slow decay with a structural relaxation time (*τ*_0_) of ≈1.3 ps. In contrast, the D_80_‐gel exhibits a much faster initial decay within the first 0.5 ps, with a local time constant (*τ*_1_) of ∼0.15 ps, suggesting the presence of transient hydrogen bonds that rapidly break and reform. Following this rapid decay, the D_80_‐gel curve quickly stabilizes at a higher plateau, unlike the continuous decline observed in the original gel. This biphasic behavior indicates a coexistence of short‐lived dynamic bonds and a persistent hydrogen‐bonding network in the D_80_‐gel, reflecting both fast reorganization and structural resilience. The comprehensive evidence from both DFT and MD simulations elucidates how the crowded and competitive hydrogen bond environment in the D_80_‐gel facilitates ultrafast structural reorganization, leading to the observed macroscopically low‐hysteresis behavior.

### 2.4 Electrical and Sensing Performance

As a flexible sensor material, D‐gels not only possess extremely low hysteresis and high mechanical performance but also demonstrate high sensitivity, ultra‐fast response, and excellent electrical stability. Free anions and cations attach to the polymer chains and migrate in opposite directions (toward the anode and cathode, respectively), forming conductive pathways and achieving a conductivity of 0.4–0.9 S cm^−1^ (Figure S17, Supporting Information). Zwitterions derived from SBMA minimize counterion condensation and promote continuous ion‐hopping transport.^[^ ^59^, ^60^ ^]^ Conductivity tests on C*_x_*‐gels (Figure S18, Supporting Information) show a sharp drop (0.9 to 0.05 S cm⁻¹) with increased glycerol content, due to glycerol's viscosity and dielectric shielding hindering ion mobility. In contrast, SBMA/glycerol‐based D*_x_*‐gels maintain stable conductivity, likely due to zwitterions’ intramolecular charge redistribution and strong electrostatic and hydrogen bonding interactions, which preserve localized ionic pathways even at high glycerol loadings.

**Figure 5:**
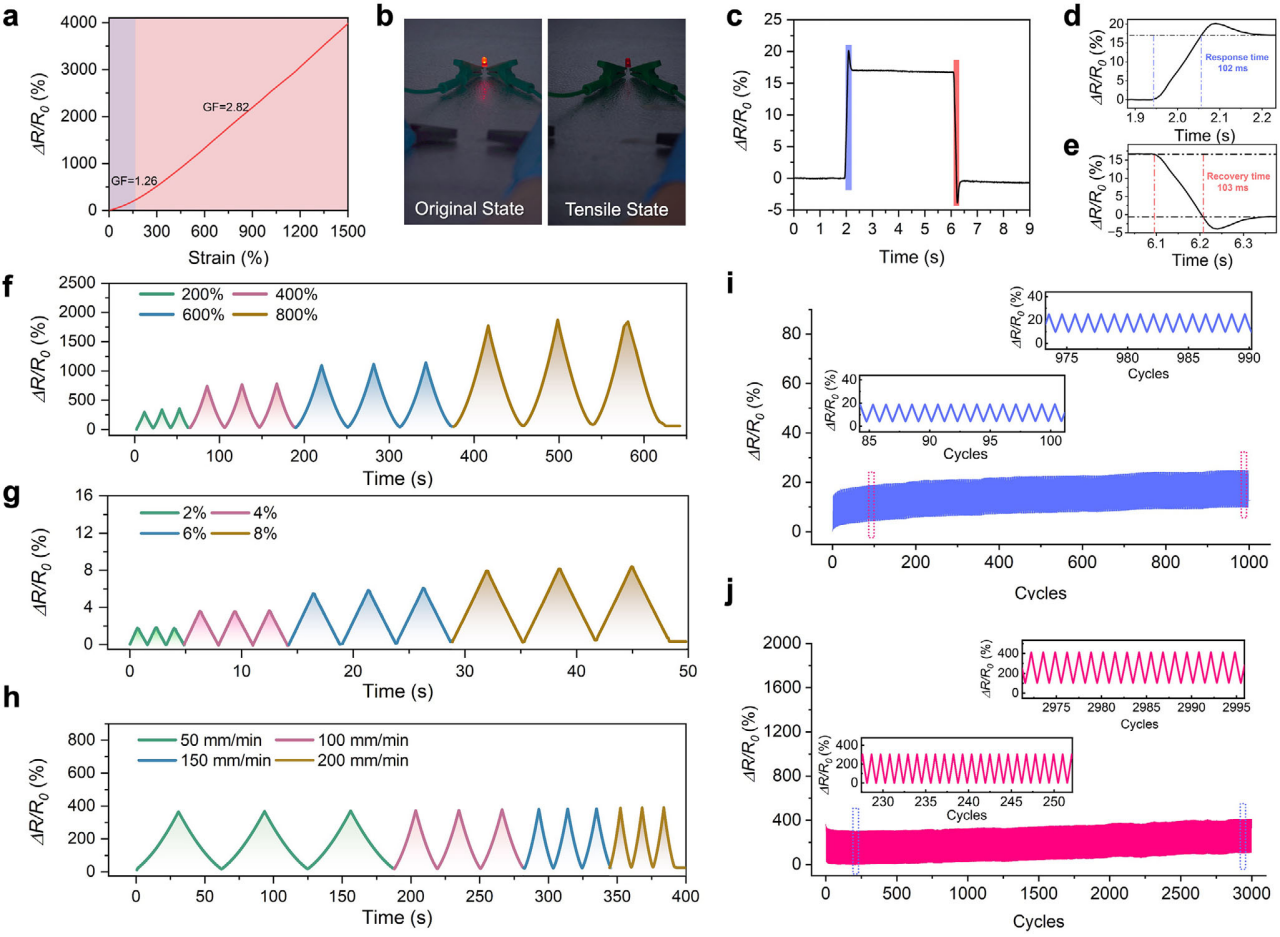
Electrical properties and sensing performance of low hysteresis D_80_‐gels. a) Relative resistance change (*ΔR/R*_0_) as a function of strain, showing gauge factors (GF) of 1.26 and 2.82 at low and high strain regimes, respectively. b) Demonstration of the gel's conductive behavior in its original and tensile states by illuminating a connected LED bulb. c) Time‐resolved response and recovery of the gel during stretching cycles. Blue and red segments indicate response and recovery phases, respectively. d) Zoomed‐in view of the response time, showing a fast response of 102 ms. e) Zoomed‐in view of the recovery time, demonstrating a recovery time of 103 ms. f) Relative resistance change (*ΔR/R*_0_) under large cyclic strains (200%, 400%, 600%, and 800%) at a constant stretching speed. g) Relative resistance change (*ΔR/R*_0_) under small cyclic strains (2%, 4%, 6%, and 8%), displaying high sensitivity and linearity at low strain levels. h) Relative resistance change (*ΔR/R*_0_) at different stretching speeds (50, 100, 150, and 200 mm min^−1^) at a constant strain of 200%, illustrating rate‐dependent electrical behavior. i) Cyclic stability test of the gel under 1000 cycles of 20% strain. j) Cyclic stability test under 3000 cycles of 200% strain.

Conductive hydrogels typically show changes in impedance when deformed, while maintaining consistent and repeatable electrical properties during deformation is crucial for their effective use in sensing applications.^[^ ^61^, ^62^, ^63^, ^64^ ^]^ **Figure** **5**a plots the relationship between the relative change in resistance (Δ*R*/*R*_0_) and tensile strain (*ε*) for the D_80_‐gel during stretching. As the strain increases from 0% to 1500%, the continuous sliding of interpenetrating polymer chains results in network deformation, leading to a dynamic change in the carrier density within the conductive network.^[^ ^61^, ^65^ ^]^ Consequently, Δ*R*/*R*_0_ displays a linear increase with strain, particularly under large deformation conditions, indicating that this soft ionic conductor offers a wide strain sensing range and precise impedance tuning capabilities as a strain sensor. The gauge factor (GF), a key indicator of strain sensor sensitivity, is calculated to be 1.26 at the initial stage of applied strain (0–100%). As the strain exceeds 100%, the GF increases to 2.82. This soft sensor based on D_80_‐gel exhibits high sensitivity in both low and high strain regions, demonstrating its reliable applicability for sensing and monitoring purposes. To visually demonstrate the current‐controlled sensitivity, an LED was integrated into a flexible circuit made with D_80_‐gel (Figure 5b). As the tensile strain increased gradually, the elongation of the polymer network in the hydrogel lengthened the ion transport path, causing an increase in resistance and a gradual dimming of the LED bulb. Additionally, as shown in the electrical response test (Figure 5c), the D_80_‐gel exhibits an average electrical response time of 102 ms (Figure 5d), which is within the range of human reaction times (100–300 ms). This quick response time suggests that D_80_‐gel is suitable for applications requiring real‐time feedback, such as wearable sensors for human motion capture or other strain sensing environments. The recovery time is 103 ms (Figure 5e), with negligible hysteresis, ensuring that the response speed is sufficient for accurate and real‐time signal detection.^[^ ^66^, ^67^, ^68^ ^]^ As shown in Figure 5f,g, the D_80_‐gel demonstrates a clear, linear response to both small and large strains, ranging from as little as 2% to 800%. The consistent ∆*R*/*R*_0_ signals observed across three consecutive cycles indicate excellent stability and repeatability of the hydrogel during stretching and releasing cycles over a wide strain range. For conductive hydrogels with strain‐sensing capabilities, frequency dependence is another critical feature.^[^ ^69^, ^70^ ^]^ Figure 5h shows the good adaptability of the gel to various strain rates, maintaining consistent electrical responses across speeds from 50 to 200 mm min^−1^. This versatility is crucial for real‐world applications, such as wearable devices or soft robotics, where strain rates can change rapidly. The gel's ability to respond swiftly to these changes without lag or significant resistance variation highlights its reliability as a dynamic sensing material. Figure 5i,j presents the results of continuous loading‐unloading tests at fixed strains of 20% for 1000 cycles and 200% for 3000 cycles, respectively, with real‐time recording of ∆*R*/*R*_0_ signal values. D_80_‐gel exhibits minimal drift in resistance even after thousands of strain cycles, maintaining stable electrical performance at both 200% strain for 3000 cycles and 20% strain for 1000 cycles, demonstrating its long‐term reliability. When the force applied to the hydrogel is released, the signal returns to the baseline, maintaining stable amplitude and waveform throughout the test. This performance ensures long‐term, effective monitoring of daily activities in practical applications while maintaining accuracy and reliability.

Exposure to extreme temperatures often causes hydrogels to dry out or freeze, compromising their mechanical properties and conductivity, or even leading to complete failure. The D‐gel was specifically designed to maintain operational stability across a wide range of environmental conditions. The stability of D_80_‐gel under both high and low‐temperature environments was evaluated: over a 7‐day period at 25 and 60 °C, the D_80_‐gels exhibited less than 3% mass loss, whereas the original gel showed poor water retention with a substantial 50% mass loss within 24 h (Figure S19, Supporting Information). The superior anti‐drying performance of the D_80_‐gels is attributed to the strong hydrogen bonds formed between the non‐volatile, hydrophilic SBMA and glycerol, which effectively retain water molecules within the polymer network, providing long‐term stability. Specifically, the sulfobetaine groups in SBMA, with their zwitterionic nature, exhibit a high affinity for water molecules due to strong ion‐dipole interactions. These groups act as hydration centers, binding water molecules tightly and preventing their evaporation.^[^ ^71^ ^]^ Simultaneously, glycerol, with its multiple hydroxyl groups, forms extensive hydrogen bonds with both water molecules and the surrounding polymer chains. This creates a dense network of interactions that further restricts water mobility and reduces the rate of water loss.^[^ ^72^ ^]^ In low‐temperature environments, free water molecules within hydrogels can aggregate and form ice crystals, causing brittleness and reduced sensing performance. However, after exposure to −35 °C for 2 h, the D_80_‐gel remained soft and pliable, whereas the original gel became significantly hardened (Figure S19, Supporting Information). Furthermore, the D_80_‐gel retained its ability to power 15 LED lights at sub‐zero temperatures, highlighting its exceptional anti‐freezing capability. This resistance to freezing is attributed to the hydrogen bonds formed between DES components and water molecules, which prevent water molecules aggregation, reduce the formation of internal ice nuclei, and effectively lower the gel's freezing point. Glycerol, in particular, disrupts the regular hydrogen bonding network of water, hindering the formation of large, damaging ice crystals.^[^ ^73^ ^]^ The SBMA molecules further contribute by intercalating between water molecules, disrupting the crystallization process and promoting the formation of smaller, less disruptive ice nuclei.^[^ ^74^ ^]^

These exceptional anti‐drying and anti‐freezing properties greatly enhance the practical applicability of D_80_‐gel‐based sensors across diverse real‐world environments. Their ability to retain moisture and remain flexible at elevated temperatures ensures long‐term stability, making them suitable for high‐temperature or dry conditions without compromising performance. Simultaneously, their freezing resistance ensures reliable functionality in extremely cold environments, making them ideal for outdoor applications, wearable devices, environmental monitoring, and soft robotics designed for extreme conditions. This unique combination of properties extends the operational lifetime and durability of the sensors, ensuring accurate and consistent data collection even in harsh or fluctuating conditions.

## 3 Conclusion

In this study, we demonstrated a novel approach to achieving low‐hysteresis, high‐performance hydrogels by leveraging a dense and competitive dynamic hydrogen bonding network. The introduction of DES components created a crowded and dynamic environment that significantly altered the hydrogen bonding landscape within the D‐gel system. This reconfiguration facilitated the formation of a diverse array of hydrogen bonds with optimized strengths and rapid hydrogen bond reconstruction capabilities, enabling efficient energy dissipation and achieving near‐zero hysteresis (<3%) under extensive deformations. Our D‐gels exhibit exceptional stretchability (>1500%), rapid response times (102 ms), outstanding water retention, and antifreeze properties, highlighting their immense potential for applications in wearable technology and smart materials. Moreover, the D‐gels exhibit remarkable anti‐drying and anti‐freezing properties, extending their applicability to a wide range of environments, making them versatile and resilient material for next‐generation flexible sensing technologies.

Hysteresis is a time‐dependent phenomenon closely related to the relaxation time of the material. If the breaking and reformation of dynamic bonds occur quickly enough, the system can efficiently complete bond breaking and reformation during loading and unloading, significantly reducing irreversible energy dissipation and minimizing mechanical hysteresis. This principle is not limited to hydrogen bonds; it can also be applied to other types of dynamic interactions, such as electrostatic forces, van der Waals forces, and π‐π or cation‐π interactions. By leveraging the strategy of rapid dynamic bond reorganization, it is possible to design low‐hysteresis gel‐based sensing materials tailored for a wider range of scenarios and applications, such as flexible in vivo sensors or sensors for seawater environments.

## 4 Experimental Section

### Materials

Ammonium persulphate (APS, ≥98%) and Polyethylene glycol diacrylate (PEGDA, >99%) were purchased from Macklin. Polyvinylpyrrolidone (PVP, K88‐96), acrylamide (AAm), and *N*,*N*′‐methylenebis(acrylamide) (MBAA, 98%) were supplied by Aladdin. Sulfobetaine methacrylate (SBMA, ≥99%), sodium chloride (NaCl, 99%) and glycerol (Gly, 99%) were sourced from Bide Pharmatech Ltd. Deionized water was produced using a WP‐UPT‐20 water system. (Water Purifier ultrapure, >18.2 MΩ) All reagents were used as received without further purification.

### Preparation of DES

Gly and SBMA were mixed at a mass ratio of 3:1. The mixture was then heated to 60 °C under continuous stirring until a uniform solution was obtained. The resulting viscous solution was then treated in a GA008G ultrasonic cleaner (GANBO, China) to remove any air bubbles formed during the mixing process.

### Preparation of Gels

D‐gels were synthesized via a one‐step random copolymerization process. First, AAm, PVP, and NaCl were dissolved in a DES/H_2_O mixed solvent and stirred magnetically at room temperature for 3 h. Next, cross‐linking agents (MBAA, 1 mm, and PEGDA, 1 mm) along with the thermal initiator (APS, 18 mm) were added, and the mixture was stirred for an additional 20 min. The pre‐polymerized solution was then degassed using an ultrasonic cleaner to remove any remaining trapped air bubbles. The degassed solution was transferred into glass molds using a syringe and polymerized in an LTI‐400E biochemical incubator (EYELA, Japan) at 60 °C for 40 min. Detailed formulations of all D‐gels are provided in Tables S1 (Supporting Information). As a primary control, the “original gel” formulation consisted solely of PAAm and PVP crosslinked with MBAA and PEGDA. C*_x_*‐gels, serving as control hydrogels based on conventional ChCl/glycerol DESs, were prepared using a 3:1 glycerol:ChCl DES as the solvent, with acrylamide as the monomer and MBAA as the crosslinker, followed by thermally initiated polymerization (APS, 18 mm; 60 °C for 40 min).

### Preparation of PAAm Gel

In this study, PAAm hydrogel was employed as the control group for crack propagation resistance investigations. The hydrogel was prepared by dissolving AAm monomer in deionized water to a concentration of 5.63 mol L^−1^. MBAA was added as a crosslinker (1 mm), and APS was added as an initiator (18 mm). The mixture was stirred, degassed, transferred to glass molds, and polymerized at 60 °C for 40 min.

### Material Characterizations

Fourier‐transform infrared (FTIR) spectra of the D‐gels samples were obtained using an INVENIO‐S FTIR spectrometer (Bruker, Germany) in attenuated total reflectance (ATR) mode. The hydrogels were quenched in liquid nitrogen and then freeze‐dried in an LGJ‐12D lyophilizer (THE FOURTH RING, China) for two days. The fractured surfaces of the freeze‐dried hydrogels were sputter‐coated with a thin layer of gold, and imaged using a GEMINISEM 360 scanning electron microscope (Zeiss, Germany) to examine the internal gel structure.

*Mechanical Measurements*: Tensile tests were performed using a Instron 5969 tensile tester (Instron, USA) equipped with a 50 N load cell. Unless otherwise specified, dumbbell‐shaped hydrogel samples (GB/T 528‐2009, 12 mm × 2 mm × 2 mm) were stretched uniaxially at a rate of 100 mm min^−1^. To evaluate the strain‐rate dependence of mechanical properties, additional tensile tests were conducted at varying stretching rates (50, 100, 200, 400, 800 mm min^−1^). Fracture toughness was calculated by integrating the area under the stress–strain curve from the raw data, and the elastic modulus was determined by fitting the slope of the initial linear region (5–15%) of the stress–strain curve. All measurements were performed at room temperature, with each formulation tested five times. Data are presented as mean ± standard deviation. The energy dissipation (*ΔE*) during cyclic tensile loading and unloading tests was calculated by integrating the area between the loading and unloading curves using Equation (1).

(1) $$\Delta E=\int_{loading}\sigma \left(\mu \right)d\mu -\int_{unloading}\sigma \left(\mu \right)d\mu$$

where *σ*(*μ*) denotes tensile stress and *μ* denotes strain. The areas beneath the loading and unloading curves, denoted as *W_loading_* and *W_unloading_*, respectively, were used to determine the hysteresis ratio (h) using Equation (2).

(2) $$h=1-\frac{W_{unloading}}{W_{loading}}$$

*Crack Propagation Test*: Single‐edge notched (pre‐cracked) specimens were prepared from rectangular hydrogel sheets (20 mm width × 40 mm length). An initial notch of 10 mm length was introduced using a razor blade. The specimen was clamped on two opposing sides, maintaining a gauge length of 8 mm between the clamps. Uniaxial tensile testing was performed at the speed of 10 mm min^−1^ until complete fracture of the specimen occurred. The crack propagation strain was defined as the strain value at which visible crack extension was initiated. The fracture energy was determined by integrating the area under the stress–strain curve up to the point of fracture.

### Rheological Characteristics

Rheological measurements were performed using an ARES‐G2 advanced rotational rheometer (TA, USA). Disc‐shaped samples (2 mm thick, 15 mm in diameter) were placed on 25 mm diameter plates and surrounded by silicone oil to prevent evaporation. Relaxation times were investigated using the time‐temperature superposition (TTS) principle, with frequency sweeps conducted from 0.1 to 100 rad s^−1^ at a shear strain of 0.5% over a temperature range of −50 to 30 °C. Data were processed using manual shifting for superposition and spectrum calculations.

### Conductivity Measurements

The electrical conductivity of the hydrogels was measured using an AUT52065 electrochemical workstation (Metrohm, Switzerland). Hydrogel samples (10 mm × 10 mm × 2 mm) were placed between two parallel copper electrodes, and conductivity (*σ*, S cm^−1^) was calculated using the formula: *σ* = *L*/(*R* × *S*), where *L* (cm) is the distance between the hydrogels during measurement, *R* (Ω) is the resistance of the hydrogels, and *S* (cm^2^) is the cross‐sectional area of the hydrogels. A constant voltage of 1 V was applied to the hydrogel, and the relative change in resistance due to varying strains was expressed as sensitivity. The gauge factor (GF) was calculated as: GF = [(*R* − *R*_0_)/*R*_0_]/*ε* = (*ΔR/R*_0_)/*ε*, where *R*_0_ and *R* are the resistances of the unstrained and strained hydrogels, respectively, and *ε* is the strain.

The response time of the D_80_‐gel was measured using dumbbell‐shaped samples (12 × 2 × 2 mm). The electrochemical workstation was integrated with a tensile tester, and the stretching rate was set to 800 mm min^−1^ with a displacement change of 1 mm. The response time was defined as the difference between the signal change time and the displacement change time.

### Drying Properties

Two sets of D‐gel samples were placed in ovens at 25 and 60 °C, respectively, to investigate their desiccation resistance. The rate of mass change (wt.%) over time was calculated using the equation: *w*% = (*W*_𝑡_/*W*_0_) × 100%, where *W*_0_ is the initial mass and *W*_𝑡_ is the mass at a given time point during the drying process.

## Conflict of Interest

The authors declare no conflict of interest.

## Supporting information



Supporting Information

Supplemental Video 1

Supplemental Video 2

## Data Availability

The data that support the findings of this study are available from the corresponding author upon reasonable request.
