# Supplementary material for: Ultra‐Low Hysteresis Under Large Deformation Enabled by Fast Chains Relaxation in Highly Competitive Dynamic Hydrogen Bond Networks
Source: Adv Sci (Weinh). 2025 Jun 25;12(36):e05417. doi: 10.1002/advs.202505417 (PMC12463079; doi:10.1002/advs.202505417)
Supplement: Supplementary file 1 — Supporting Information [file ADVS-12-e05417-s001.docx]

Supporting Information

Ultra-Low Hysteresis Under Large Deformation Enabled by Fast Chains Relaxation in Highly Competitive Dynamic Hydrogen Bond Networks

Shuaijun Guo^a,1^, Shilei Zhu^a,1*^, Yang Qiao^a^, Shanhao Feng^a^, Xin Yang^b^, Beibei Kang^a^, Chaojun Yue^a^, Yanjing Zhang^a^, Zhuangzhuang Li^a^, Ya Nan Ye^a*^, Qiang Zheng^c^

^a^ College of Materials Science & Engineering, Taiyuan University of Technology, Taiyuan 030024, China

^b^ Shaanxi Key Laboratory of Chemical Additives for Industry, Xi’an Key Laboratory of Advanced Performance Materials and Polymers Shaanxi University of Science and Technology, Xi’an 710021, China

^c^ Department of Polymer Science and Engineering, Zhejiang University, Hangzhou 310027, China

^1^ These authors contributed equally to this work.

^*^ E-mail: [zhushilei@tyut.edu.cn](mailto:zhushilei@tyut.edu.cn); [yeyanan@tyut.edu.cn](mailto:yeyanan@tyut.edu.cn)

Table S1. Composition details of D-gels

| Sample | AM  **(M)** | PVP  **(wt%)** | MBAA  **(mM)** | NaCl  (mg) | PEGDA  **(mM)** | APS  **(mM)** | H_2_O  **(g)** | **SBMA**  **(g)** | Gly  **(g)** |
| --- | --- | --- | --- | --- | --- | --- | --- | --- | --- |
| Original gel | 5.63 | 2.85 | 1 | 470 | 1 | 18 | 15 | 0 | 0 |
| D_20_-gel | 5.63 | 2.85 | 1 | 470 | 1 | 18 | 12 | 0.75 | 2.25 |
| D_40_-gel | 5.63 | 2.85 | 1 | 470 | 1 | 18 | 9 | 1.75 | 4.25 |
| D_60_-gel | 5.63 | 2.85 | 1 | 470 | 1 | 18 | 6 | 2.25 | 6.75 |
| D_80_-gel | 5.63 | 2.85 | 1 | 470 | 1 | 18 | 3 | 3 | 9 |

Table S2. Hysteretic properties of hydrogels prepared by different methods

| **Types of gels** | **Materials** | **Strain** | **Hysteresis** | **References** |
| --- | --- | --- | --- | --- |
| Deep Eutectic Solvent gels | PAAm/THMA/ILs | 500%-800% | 4−5.5% | [1] |
|  | PAA/Phyx | 100% | 38% | [2] |
|  | PAM/THMA/PIL | 100%-500% | 9% | [3] |
|  | PAM/HA/Zn^2+^ | 100% | 16.26% | [4] |
|  | PAAm/ChCl/U | 1100% | >30% | [5] |
| Elastic gels | PAAm | 100%, 200%, 300% | 0.2%-0.5% | [6] |
|  | PAM/CaCl_2_ | 800% | 0.13% | [7] |
| Nanoparticle–Hydrogels | PAM/LSN/Fe^3+^ | 700% | <15% | [8] |
|  | PHC/Ca^2+^/SiO_2_ | 100% | 18% | [9] |
| Sliding-ring gels | PAAM/αCDAAmMe | 1350% | 20% | [10] |
|  | PR-Alg | 1000% | 11% | [11] |
| Other gels | PVA/CBA/PAH | 200% | 5% | [12] |
|  | PVP/PAM | 300%-900% | 30% | [13] |
|  | PAAM/PAA/ZnCl_2_ | 1000% | >30% | [14] |


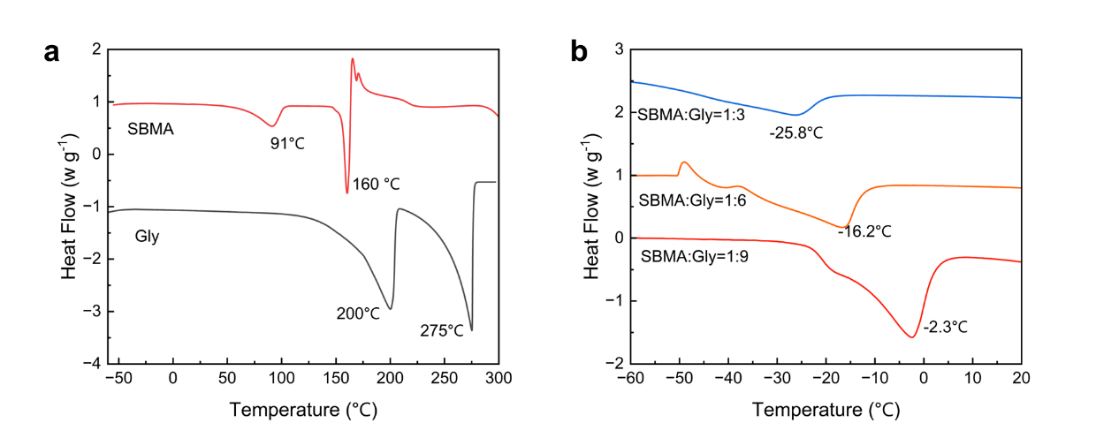


**Figure S1**. Differential scanning calorimetry (DSC) analysis of SBMA, glycerol, and their DES mixtures. a) DSC curves of pure SBMA and glycerol. b) DSC curves of SBMA/glycerol DES mixtures at different ratios, showing melting point depression.


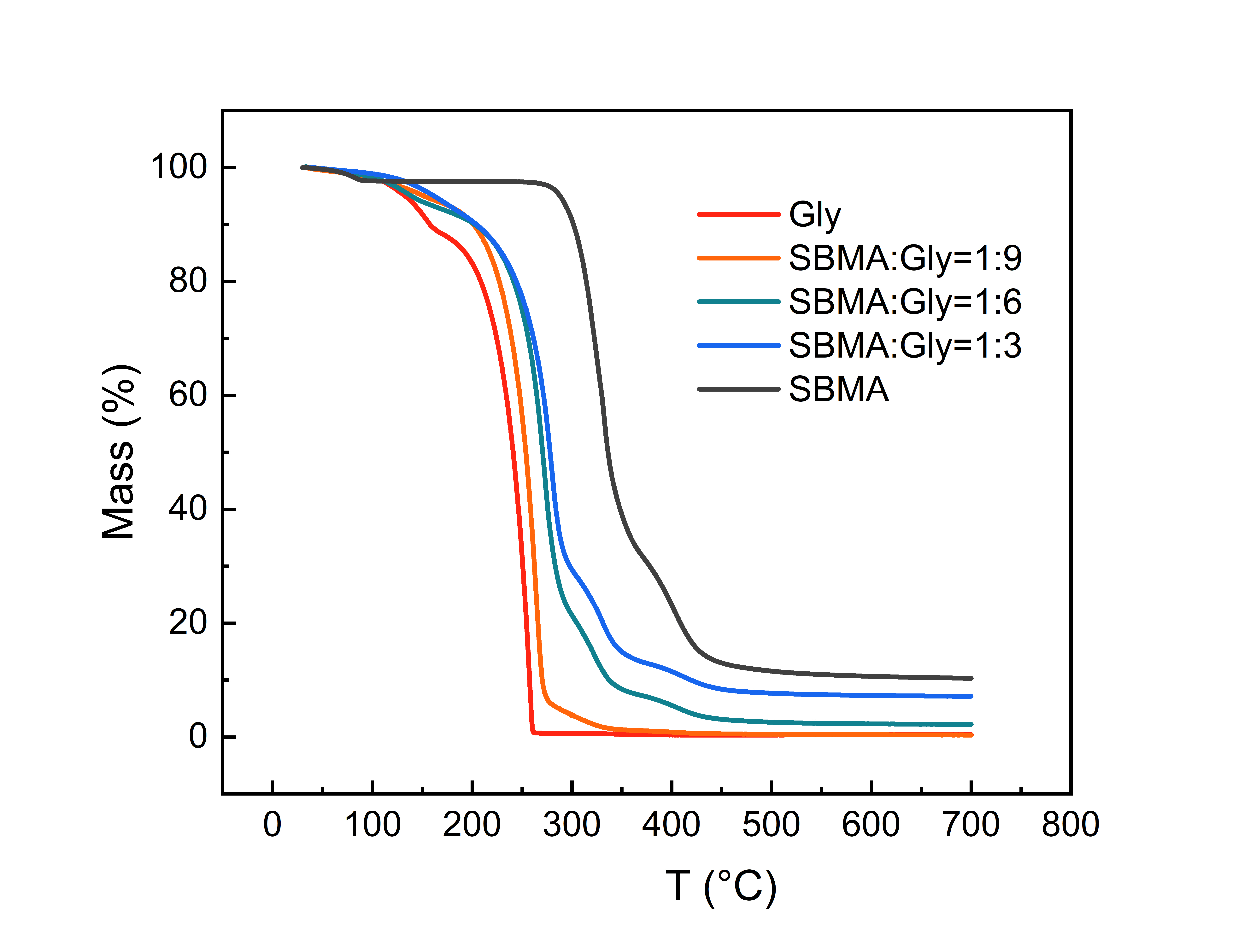


**Figure S2.** Thermogravimetric analysis (TGA) curves of pure glycerol (Gly), SBMA, and SBMA/glycerol DES mixtures with different mass ratios (SBMA:Gly = 1:9, 1:6, and 1:3), recorded over a temperature range of 30-700 °C.

**
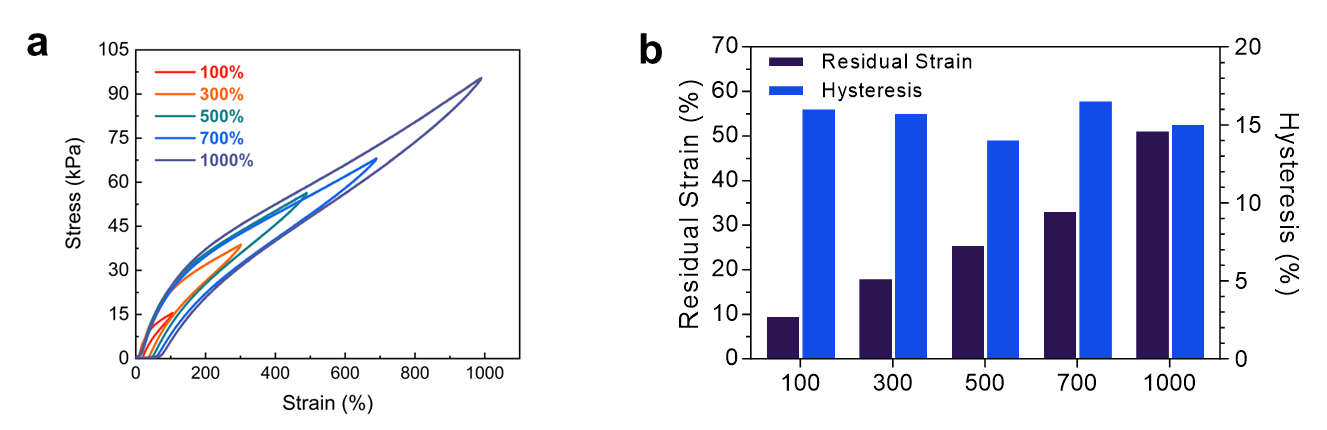
**

**Figure S3.** a) Stress-strain curves of glycerol-only gels at various strain levels (100%, 300%, 500%, 700%, and 1000%). b) Hysteresis and residual strain for glycerol-only gels at different strain levels.

**DFT Computational Details**

All quantum chemical calculations were performed using the Gaussian 16 program suite.^[15]^ Electronic structure optimizations and analyses were conducted within the framework of Density Functional Theory (DFT). The ground-state geometries of all studied molecules and complexes were fully optimized using the B3LYP-D3BJ functional, which incorporates the Becke three-parameter hybrid exchange-correlation functional (B3LYP) augmented with the D3 version of Grimme’s dispersion correction (BJ damping) ^[16]^ to account for long-range van der Waals interactions. The 6-311G(d) basis set was employed for all atoms, providing a balance between computational cost and accuracy for systems of this size. The solvent effect was included in the calculations using the solvation model based on the density (SMD) model.^[17]^ The vibrational frequencies of the optimized structures were carried out at the same level. The structures were characterized as a local energy minimum on the potential energy surface by verifying that all the vibrational frequencies were real. The absence of imaginary frequencies confirmed the thermodynamic stability of the structures. Different binding modes were explored, and their stability was assessed based on interaction energy and geometric optimization. The interaction energy (ΔE) of the optimized complexes was calculated. ΔE is defined as the difference between the complex and the sum of energies of monomers, which can be obtained by the following formulas ΔE =E*_complex_*-E*_monomerA_*-E*_monomerB_*. The Atoms in Molecules (AIM) analysis and the independent gradient model (IGM) analysis was derived by using the Multiwfn software_._^[18]^ The IGM isosurfaces were visualized by the Visual Molecular Dynamics (VMD) program.^[19]^


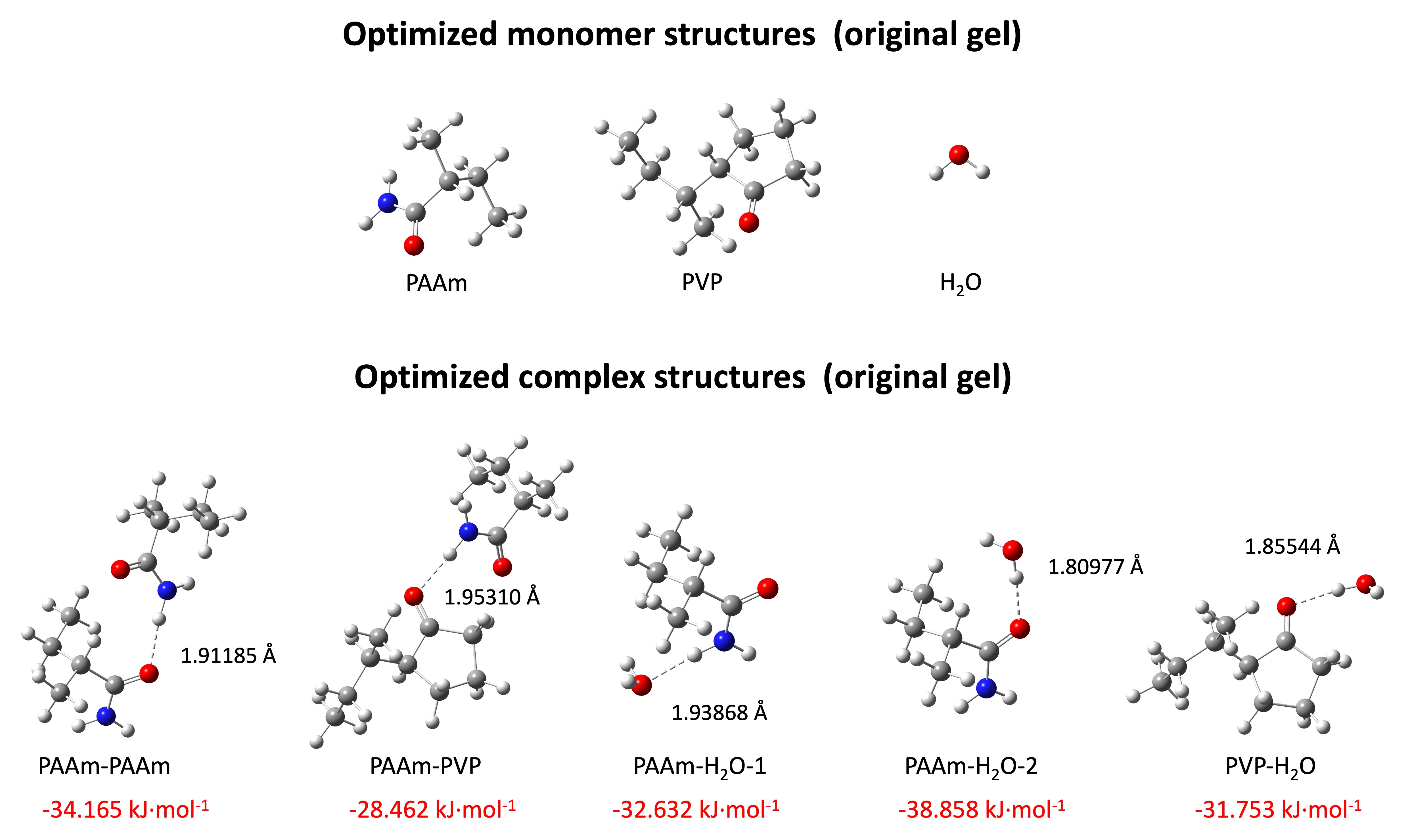


Figure S4. The optimized structures of all monomers and their corresponding hydrogen bonding complexes in the original gel, as well as the hydrogen bonding energies of the corresponding complex structures.


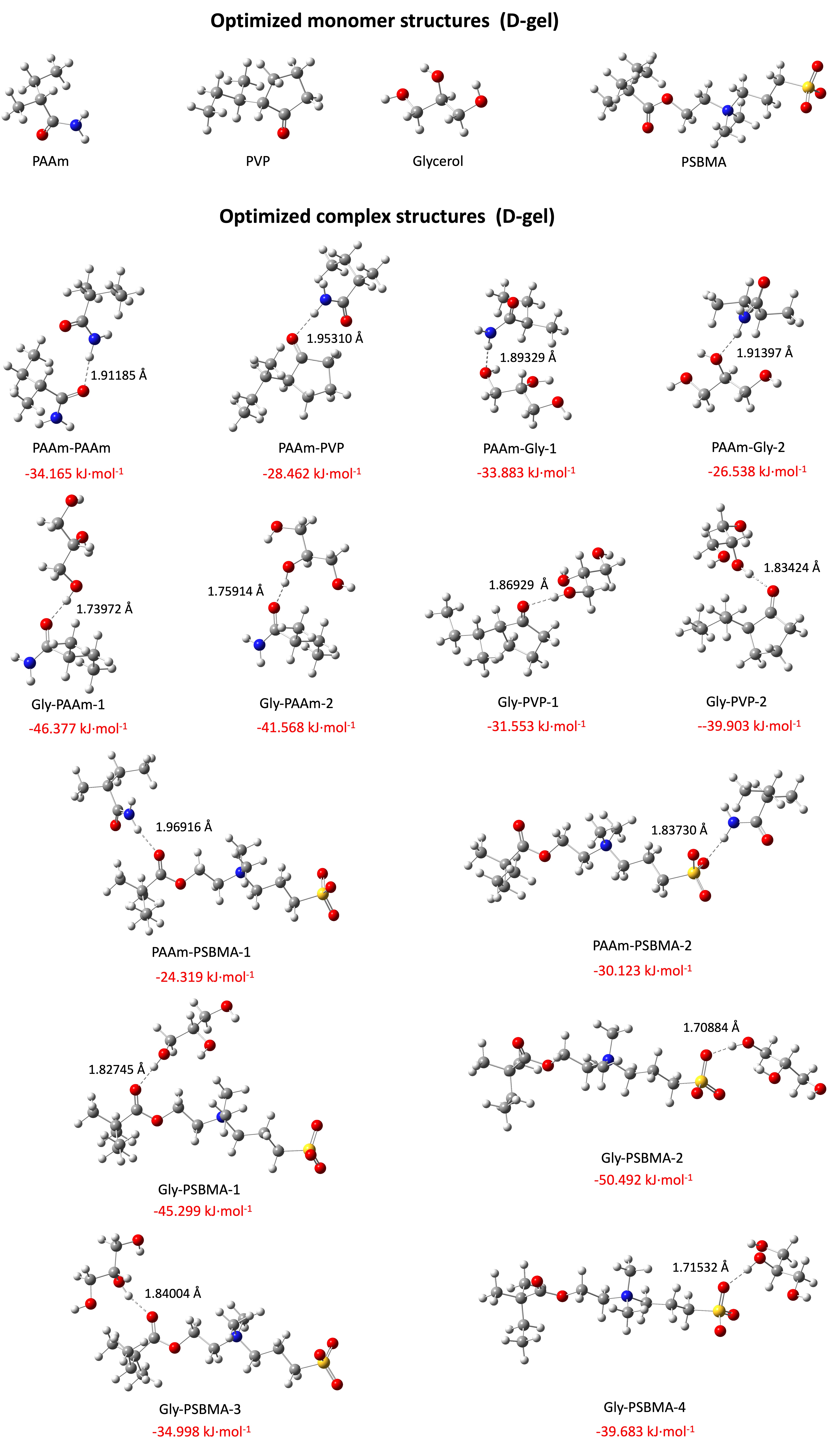


Figure S5. The optimized structures of all monomers and their corresponding hydrogen bonding complexes in the D-gel, as well as the hydrogen bonding energies of the corresponding complex structures.


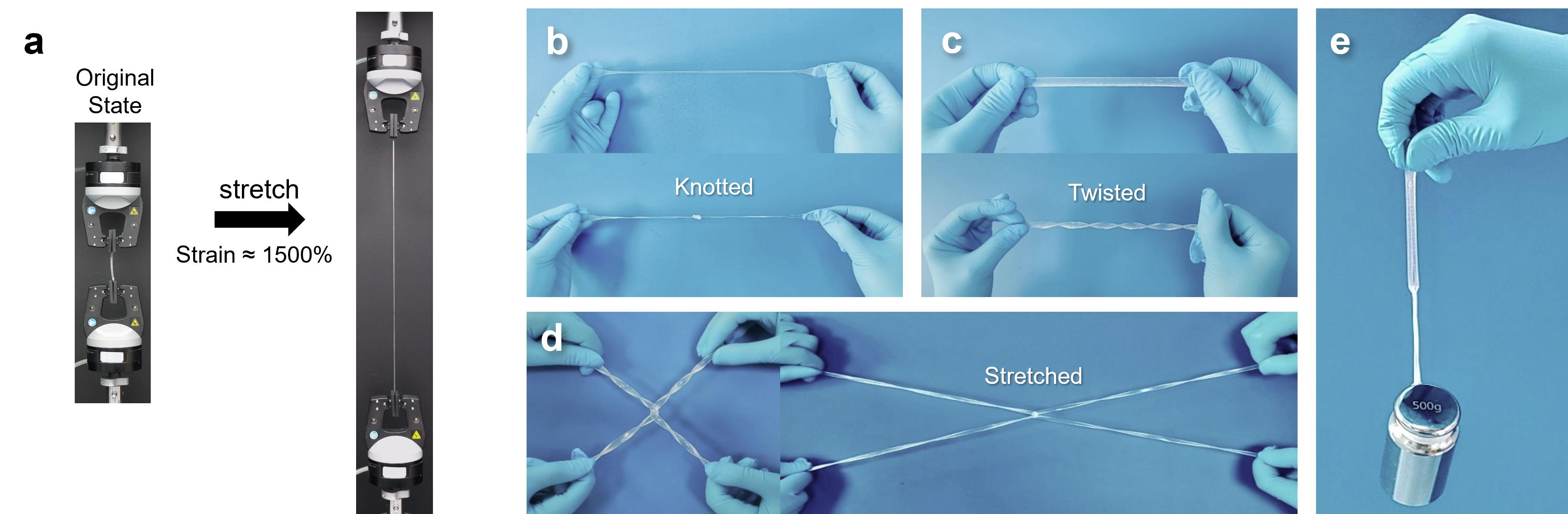


Figure S6. a) Photographs of the D_80_-gel hydrogels exhibiting excellent mechanical properties (strain = 1500%). b) Stretching, and knotting stretching of dumbbell-shaped D-gels. c) Twisting stretching of strip hydrogel. d) Biaxial twist stretching and e) lifting capability of striped D-gels.


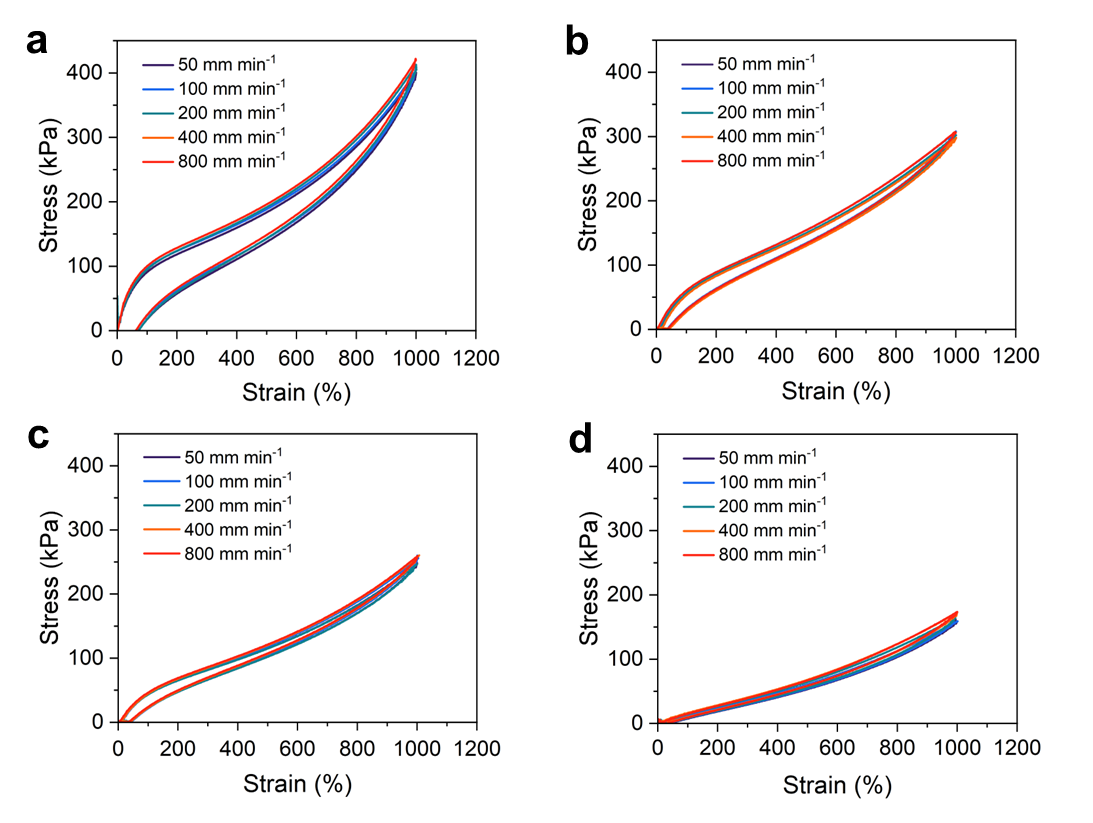


Figure S7. The stress-strain curves of different gels at 1000% strain under various tensile speeds (50-800 mm·min⁻^1^) demonstrate minimal dependence on the tensile rate, with nearly identical curves suggesting low sensitivity to strain rate. a) original gel; b) D_20_-gel; c) D_40_-gel; d) D_60_-gel.


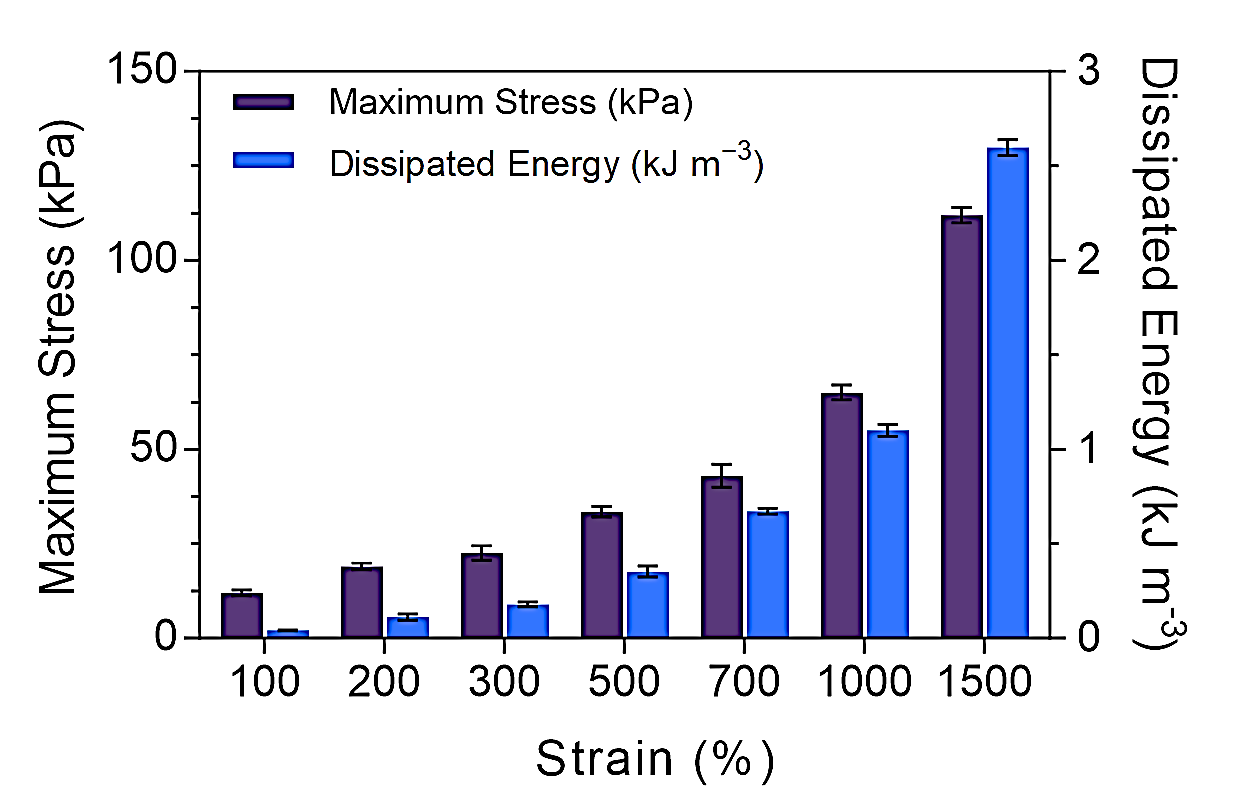


Figure S8. The graph illustrates the correlation between strain and both maximum stress and dissipated energy for D_80_-gels. With an increase in strain from 100% to 1500%, both maximum stress and dissipated energy exhibit significant growth, although dissipated energy remains relatively low at higher strain levels.


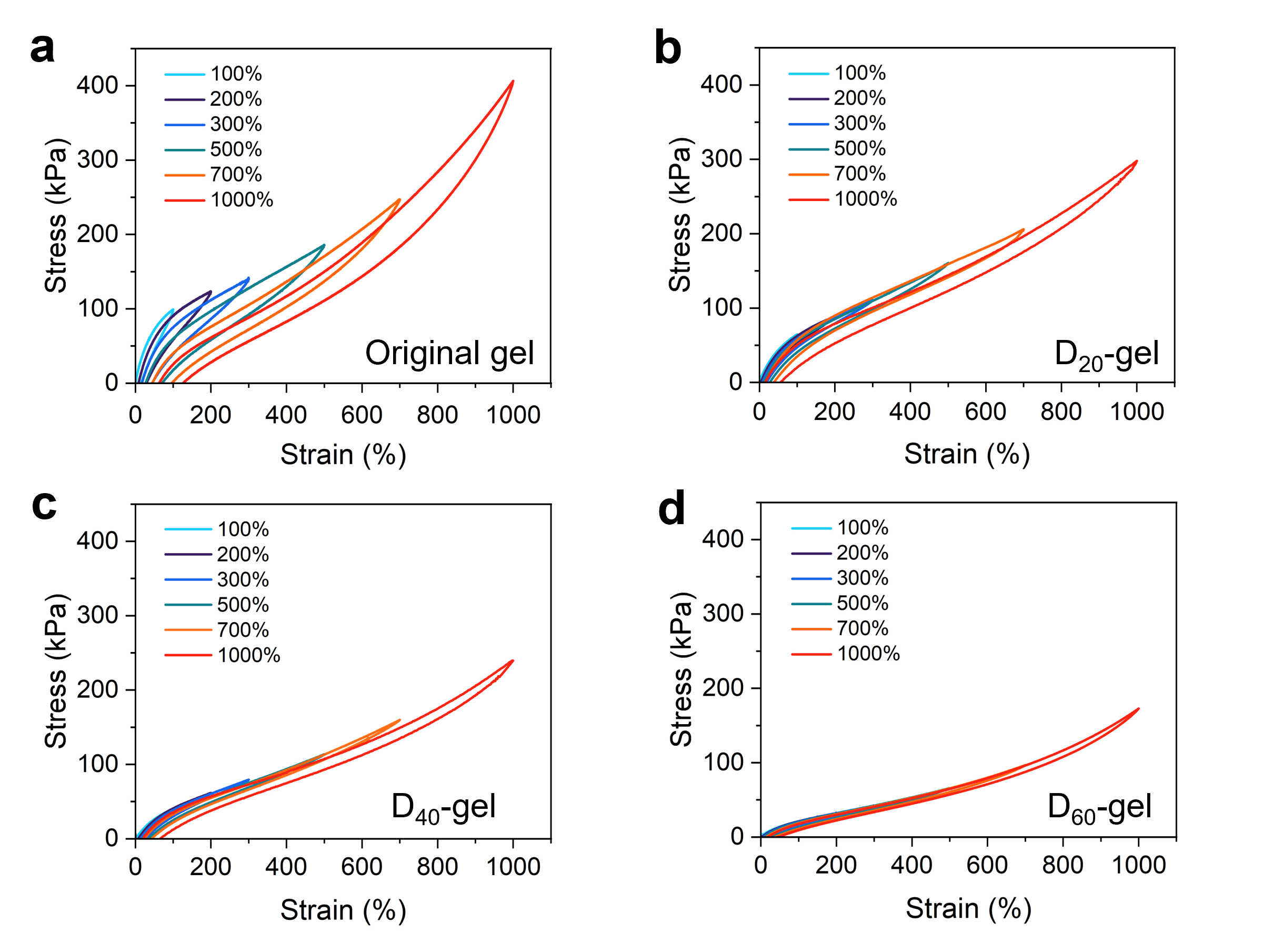


Figure S9. The stress-strain curves of various gels under increasing cyclic strains (100%–1000%) at a stretching rate of 100 mm·min⁻^1^ demonstrate that the gels maintain structural integrity throughout the process. Notably, the hysteresis loops progressively shrink with increasing DES content, reflecting a decreasing hysteresis rate. a) original gel; b) D_20_-gel; c) D_40_-gel; d) D_60_-gel.


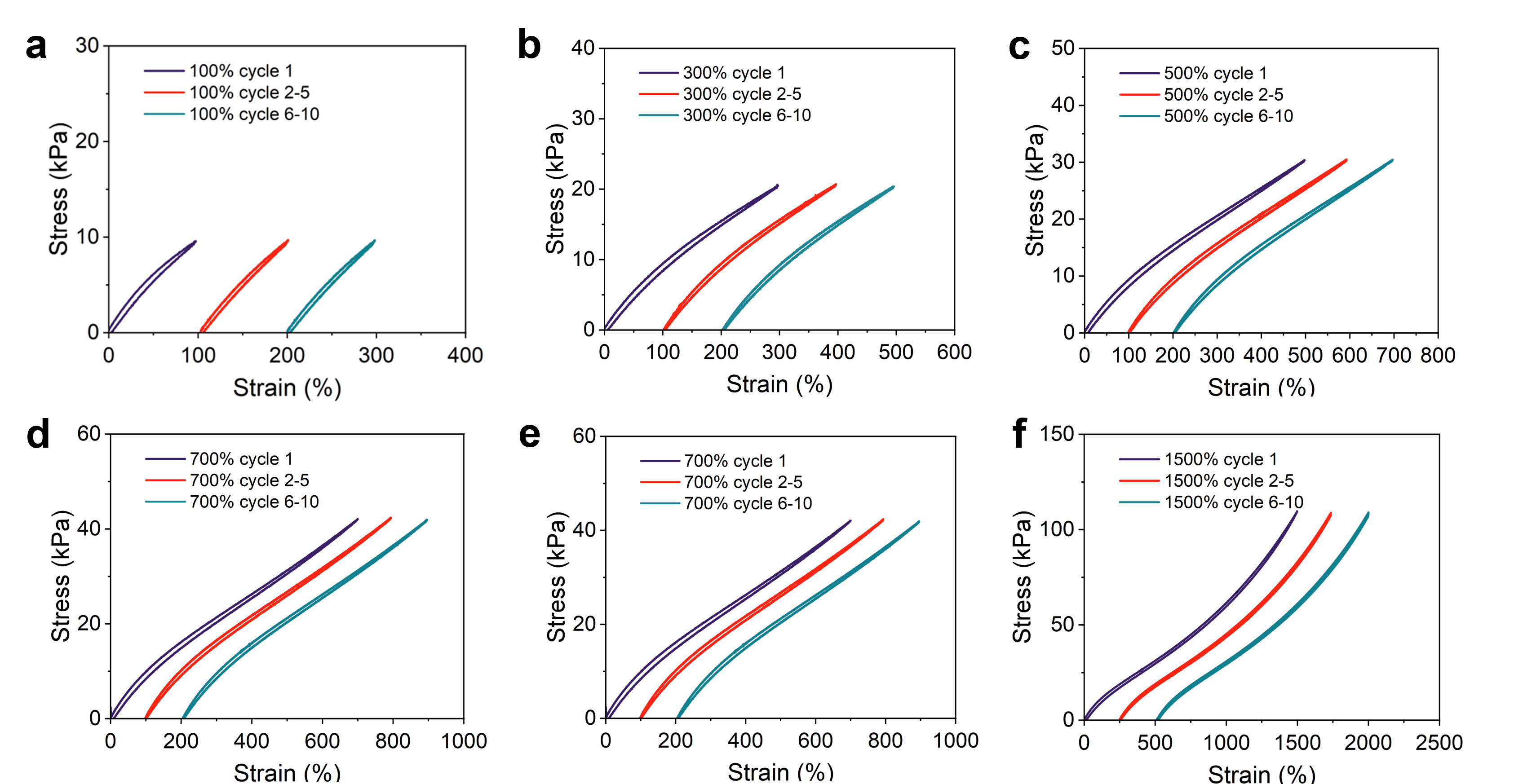


Figure S10. Stress-strain curves of D_80_-gel under multiple tensile cycles at varying strain levels demonstrate high mechanical stability and reliability. Each set of cyclic tests shows highly similar curves across cycles, indicating the gel’s excellent resilience and consistent performance under repeated loading conditions. a) 100%; b) 300%; c) 500 %; d) 700 %; e) 1000 %; f) 1500%.


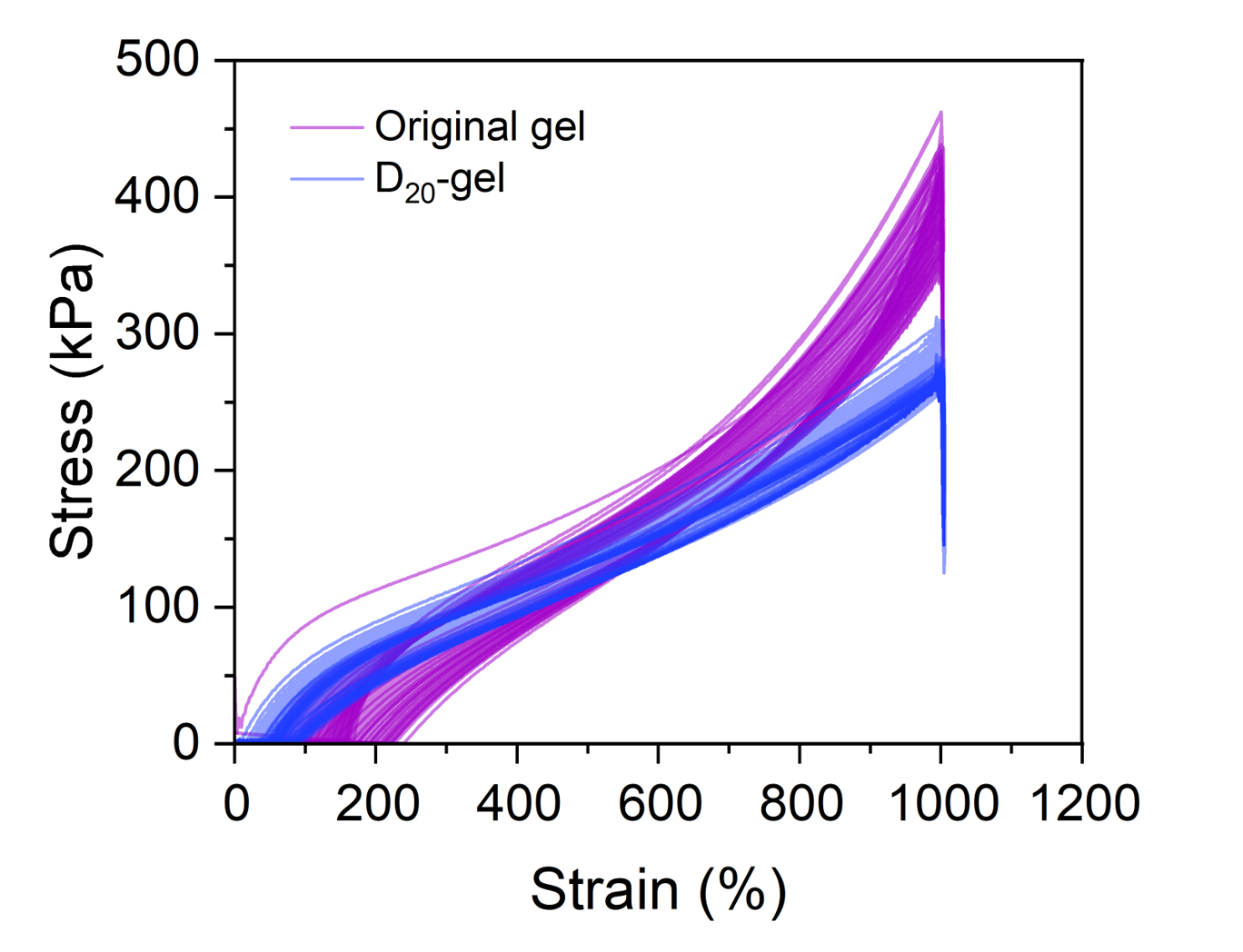


Figure S11. The stress-strain curves of the original gel and D_20_-gel were recorded over 200 continuous tensile cycles at a fixed strain of 1000% and a stretching rate of 400 mm·min⁻^1^.

(Notably, due to water loss from the hydrogel during tensile testing, the stress values in the stress-strain curves increase as the water content decreases.)


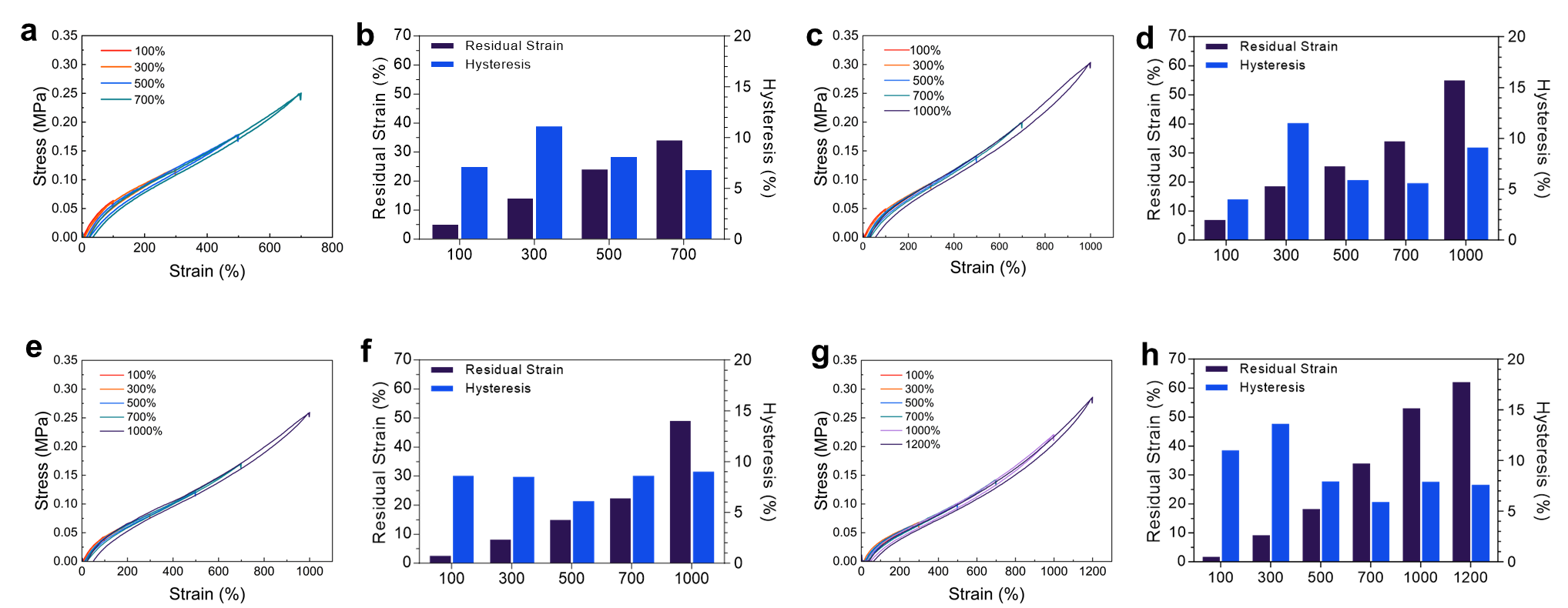


**Figure S12.** Mechanical performance and hysteresis behavior of C*_x_*-gels at various strain levels. a), b) C_20_-gel; c), d) C_40_-gel; e), f) C_60_-gel; g), h) C_80_-gel. a), c), e), g) Stress-strain curves under increasing maximum strain levels. b), d), f), h) Corresponding residual strain and hysteresis rate extracted from cyclic tensile tests.


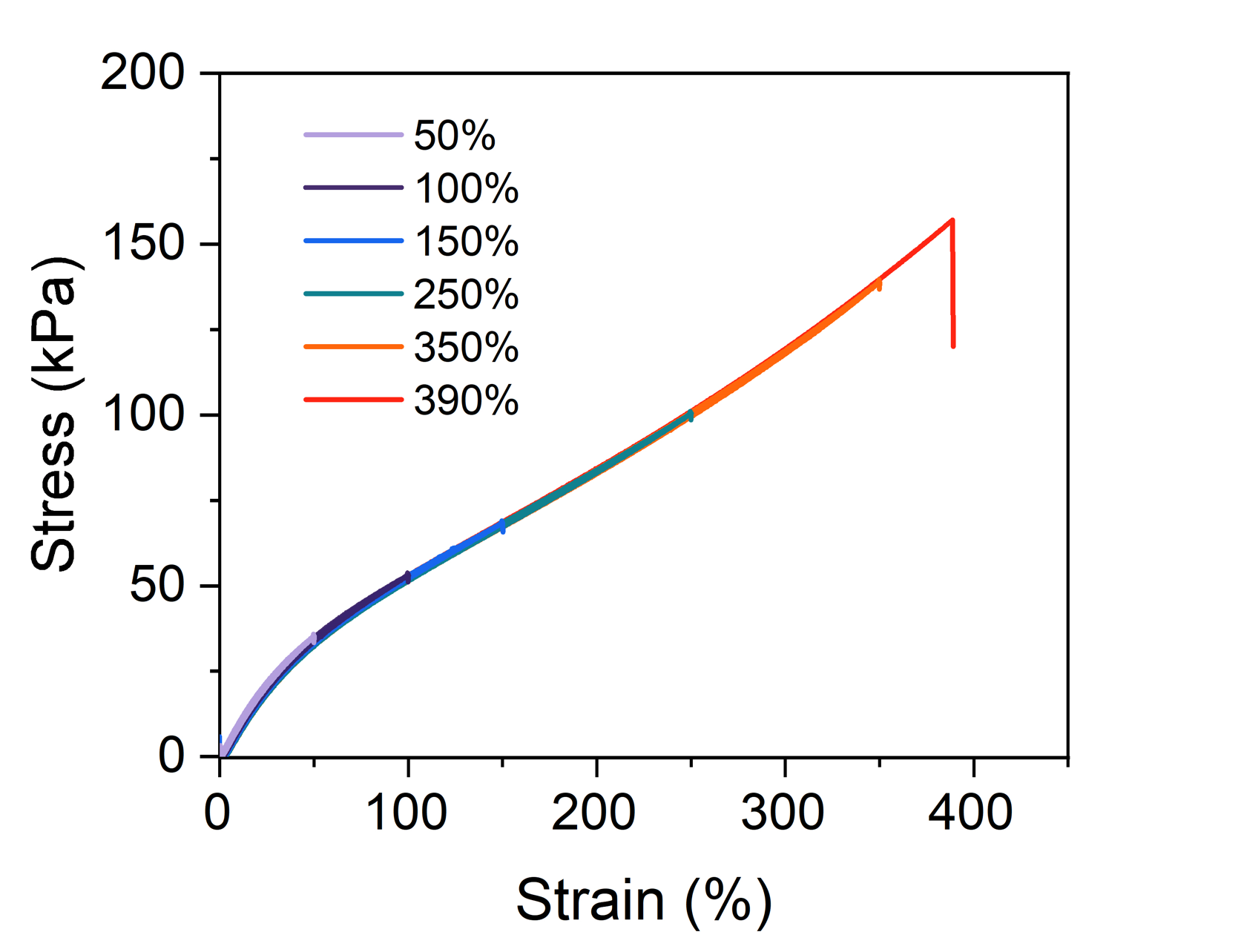


Figure S13. Stress-strain curves of PAAM hydrogels during loading and unloading under different fixed strains (50%–390%). The hydrogels exhibit purely elastic behavior with negligible internal friction and energy dissipation, resulting in almost no mechanical hysteresis during the loading-unloading cycles.

、
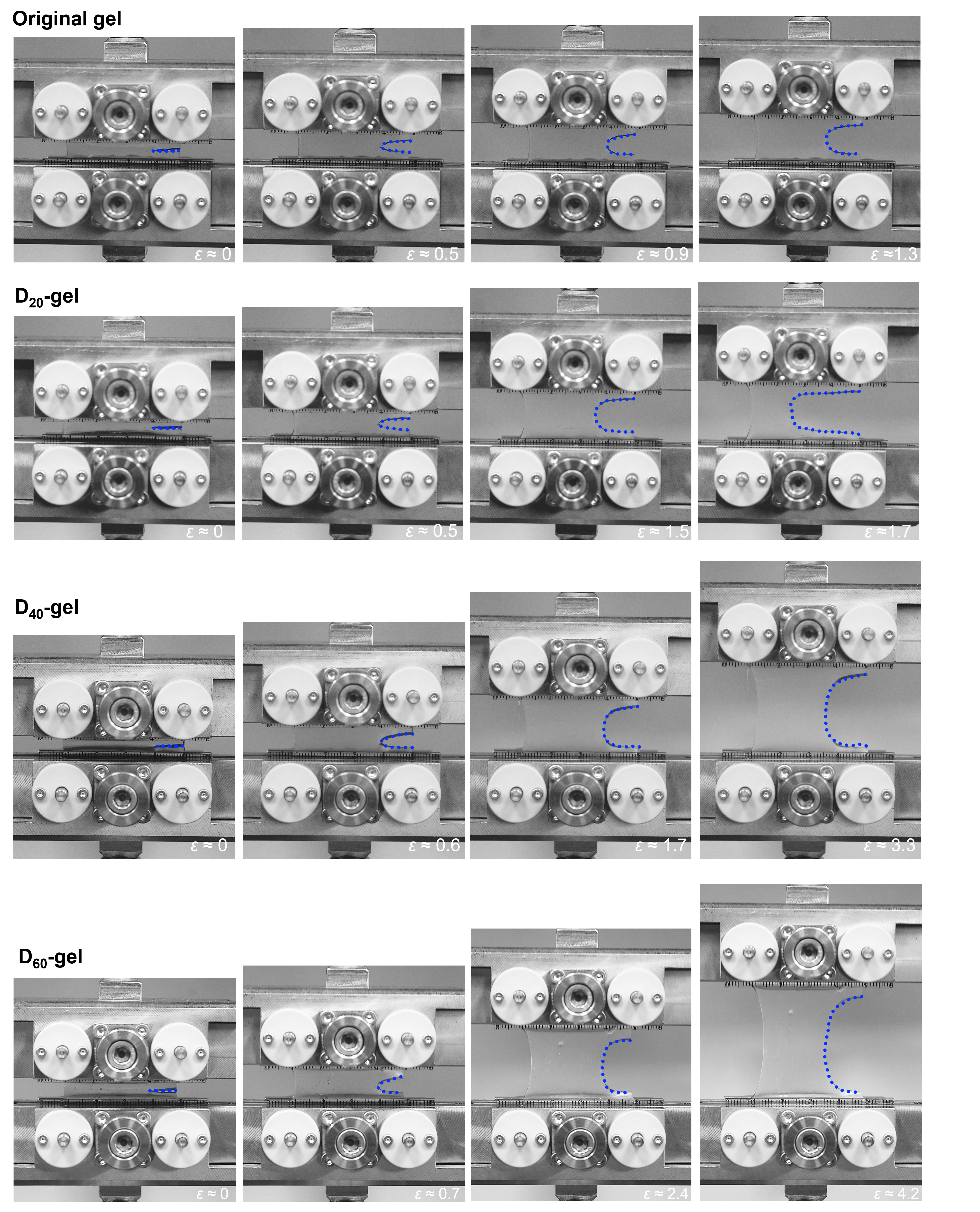


Figure S14. Sequential images showing crack propagation in D-gels with various compositions under applied strain. The results demonstrate that D-gels exhibit excellent crack propagation resistance across a wide range of compositions.


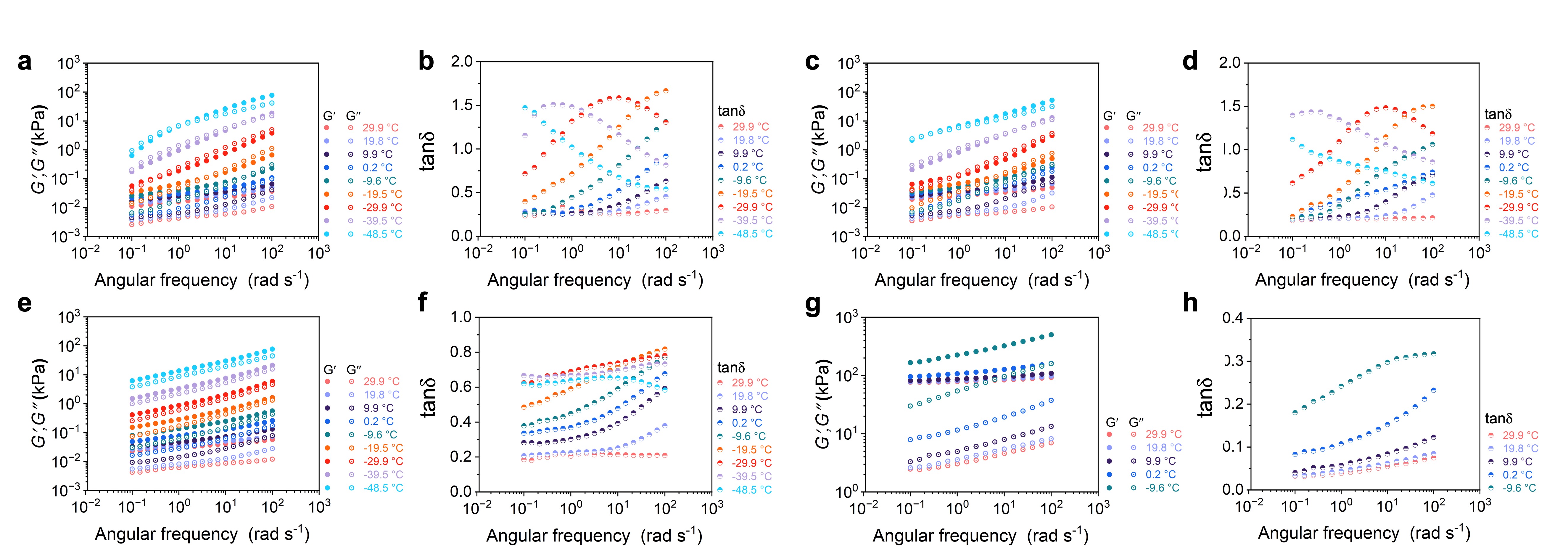


Figure S15. Dynamic mechanical properties of hydrogels at different temperatures. Plots show a);c); e); g) storage modulus (*G'*) and loss modulus (*G"*) as a function of angular frequency and b); d); f); h) loss factor (tan*δ*) as a function of angular frequency for hydrogels a), b) D_80_-gel; c), d) D_60_-gel; e) f) D_40_-gel; g), h) original gel.

**Molecular Dynamics Simulation**

In this study, we constructed PAAM molecules, PVP molecules and PSNMA molecules with a degree of polymerization of 5 in Materials Studio. Meanwhile, a glycerol molecule model was also constructed. Use Gaussian 16 code^[20]^ to optimize the structures of molecules using the B3LYP-D3 functional and the 6-31+G (d) basis set. Then Multiwfn was used to fit the restrained electrostatic potential (RESP) charge.^[21,22]^ Other bond and non-bond parameters were obtained via AuToFF software.^[23]^

All-atom molecular dynamics (MD) simulations were performed using GROMACS 2021.5. ^[24–26]^ The systems were modeled with the Amber03 force field^[27]^ and solvated in SPCE water. Two distinct systems were constructed in a 10 nm × 10 nm × 10 nm cubic simulation box: System 1 contained 170 PAAM, 10 PVP, and 1670 water molecules, while System 2 comprised 170 PAAM, 10 PVP, 20 PSBMA, 330 glycerol, and 190 water molecules.

The molecular mechanics force field incorporated both non-bonded and bonded interactions. Non-bonded interactions included van der Waals (vdW) and electrostatic terms, as described by Eqs. S1 and S2 (Supporting Information), respectively. The simulations were carried out separately for each system under identical computational conditions.

$$E_{LJ}\left( r_{ij} \right)=4\varepsilon_{ij}\left( \left( \frac{\sigma_{ij}}{r_{ij}} \right)^{12}-\left( \frac{\sigma_{ij}}{r_{ij}} \right)^{6} \right)$$

(S1)

Here, $r_{ij}$represents the distance between atoms i and j, $\varepsilon_{ij}$ denotes the energy parameter between atoms i and j, and $\sigma_{ij}$ denotes the atomic size parameter between atoms i and j.

$$E_{c}\left( r_{ij} \right)=\frac{q_{i}q_{j}}{4\pi\varepsilon_{o}\varepsilon_{r}r_{ij}}$$

(S2)

Here, $r_{ij}$ represents the distance between atoms *i* and *j*; $q_{i}$ and $q_{j}$ represent the atomic charges of atoms *i* and *j*, respectively; $\varepsilon_{o}$ is the vacuum dielectric constant; and $\varepsilon_{r}$ is the relative dielectric constant.

Nonbonded interactions were computed with a cutoff of 1.2 nm, and long-range electrostatic interactions were computed using the particle mesh Ewald (PME) method. All hydrogen bonds were constrained using the LINCS algorithm.^[28]^

In the simulation, Energy minimization was initially carried out for system, employing the steepest descent method to address initial contact issues. Followed by a short simulation of 10 ns using the NPT system to make the box fully compressed. Subsequently, A 100 ns simulation was then performed under the NVT ensemble to relax the structure and achieve system equilibrium. The pressure was maintained at P = 1.0 bar using a Berendsen barostat, and the temperature was controlled at 298.15 K using a velocity-rescale thermostat with a coupling constant of τ = 0.1 ps. Simulations were performed with a time step of 2 fs, and the neighbor list was updated every 10 steps. Periodic boundary conditions were applied in all three directions. PyMOL-3.0.3 was used for visualization.


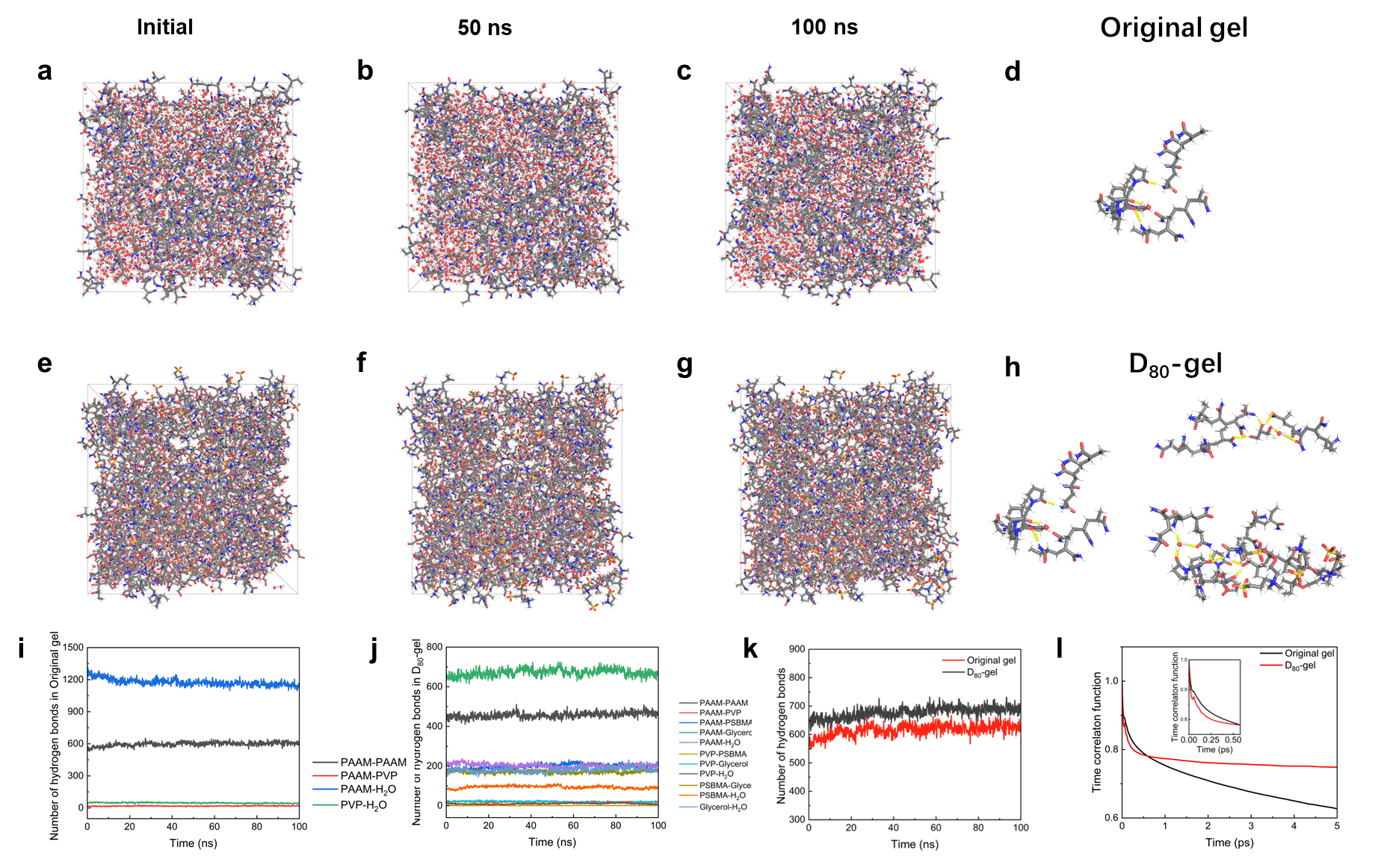


**Figure S16.** Molecular dynamics simulations and hydrogen bond analysis of two hydrogel systems. a-c) Snapshots of the original gel system at different time points during molecular simulation, revealing its structural evolution. e-g) Snapshots of the D_80_-gel system at different time points during molecular simulation, showcasing its distinct structural features. d, h) Magnified views of representative hydrogen bond types within the original gel and D_80_-gel systems, respectively, where and yellow dashed lines highlight key hydrogen bond interactions. i) Time-dependent curves of various hydrogen bond counts in the original gel system, quantifying the hydrogen bond stability between different components. j) Time-dependent curves of various hydrogen bond counts in the D_80_-gel system, revealing its more complex and dynamic hydrogen bond network. k) number of inter-chain hydrogen bonds l) Time correlation functions *C(t)* of hydrogen bond lifetimes in both original gel and D_80_-gel systems, reflecting their hydrogen bond formation and dissociation kinetics. Inset: magnified view of the green shaded area in panel k, specifically illustrating the hydrogen bond decay behavior at extremely short timescales (0-0.5 ps).


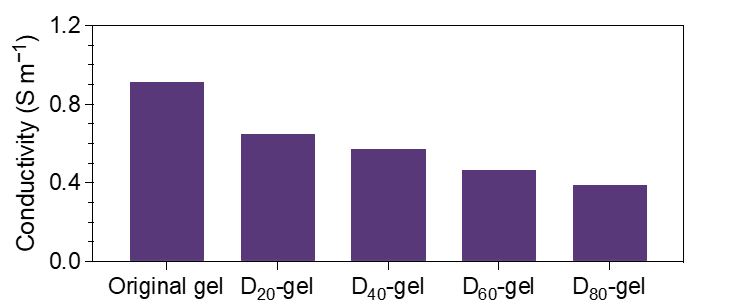


Figure S17. Electrical conductivity of hydrogels prepared with varying ratios of DES content. The plot shows the conductivity values of the hydrogels, which range from 0.4 to 0.9 S cm^−1^, depending on the DES content.

**Figure S18.** Electrical conductivity of C*_x_*-gels prepared with varying DES contents.


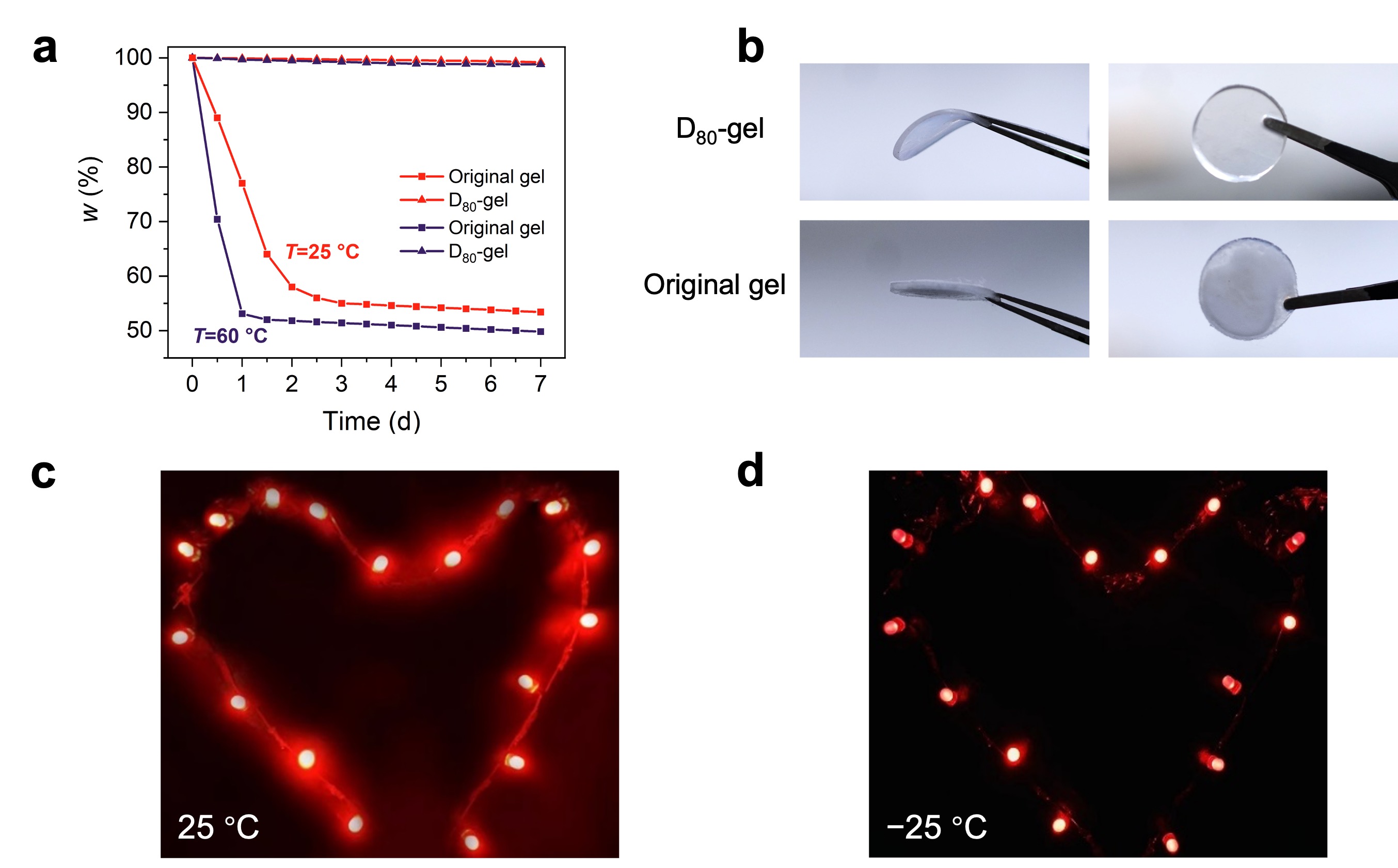


Figure S19. Water retention, anti-freezing capability, and optical transparency of D_80_-gel hydrogel. a) Water retention capacity of both the original hydrogel and D_80_-gel at elevated (60 °C) and ambient (25 °C) temperatures over a period of 7 days. b) Photographic comparison of the original gel and D_80_-gel after exposure to ambient temperature and −35 °C for 2 hours, highlighting the enhanced transparency and frost resistance of D_80_-gel. c, d) Illumination of LED lights through D_80_-gel in ambient (25 °C) and sub-zero (−25 °C) conditions, demonstrating the conductivity and stability of D_80_-gel across varied temperatures.

**References**

[1] W. Wang, P. Guo, X. Liu, M. Chen, J. Li, Z. Hu, G. Li, Q. Chang, K. Shi, X. Wang, K. Lei, *Adv. Funct. Mater.* **2024**, *34*(32), 2316346.

[2] H. Qiu, P. Guo, Y. Guan, B. Yuan, H. Li, H. Wang, R. Liu, H. Wang, R. Zhang, H. Fu, W. Cui, *Colloids Surf. A Physicochem. Eng. Asp.* **2024**, *686*, 133346.

[3] S. Han, Y. Hu, J. Wei, S. Li, P. Yang, H. Mi, C. Liu, C. Shen, *Adv. Funct. Mater.* **2024**, *34*(32), 2401607.

[4] S. Guan, C. Xu, X. Dong, M. Qi, *J. Mater. Chem. A* **2023**, *11*(28), 15404–15415.

[5] S. Li, Y. Jiang, Y. Zhu, J. Fu, S. Yan, *Colloid Polym. Sci.* **2023**, *301*(1), 19–29.

[6] J. Liu, X. Chen, B. Sun, H. Guo, Y. Guo, S. Zhang, R. Tao, Q. Yang, J. Tang, *J. Mater. Chem. A* **2022**, *10*(48), 25564–25574.

[7] J. Wang, F. Tang, C. Yao, L. Li, *Adv. Funct. Mater.* **2023**, *33*(23), 2214935.

[8] H. Zhao, S. Hao, Q. Fu, X. Zhang, L. Meng, F. Xu, J. Yang, *Chem. Mater.* **2022**, *34*(11), 5258–5272.

[9] X. Xu, C. He, F. Luo, H. Wang, Z. Peng, *Nanomaterials* **2021**, *11*(7), 1854.

[10] L. Feng, S.-S. Jia, Y. Chen, Y. Liu, *Chem. Eur. J.* **2020**, *26*(62), 14080–14084.

[11] Y. Liu, Y. Zhang, Z. An, H. Zhao, L. Zhang, Y. Cao, M. Mansoorianfar, X. Liu, R. Pei, *ACS Appl. Bio Mater.* **2021**, *4*(12), 8597–8606.

[12] T. Li, X. Li, J. Yang, H. Sun, J. Sun, *Adv. Mater.* **2023**, 2307990.

[13] Q.-J. Xu, Z.-Y. Yuan, C.-C. Wang, H. Liang, Y. Shi, H.-T. Wu, H. Xu, J. Zheng, J.-R. Wu, *Chin. J. Polym. Sci.* **2024**, *42*(5), 591–603.

[14] X. Zhao, H. Wang, J. Luo, G. Ren, J. Wang, Y. Chen, P. Jia, *ACS Appl. Polym. Mater.* **2022**, *4*(3), 1784–1793.

[15] M. J. Frisch, G. W. Trucks, H. B. Schlegel, Gaussian 16 Revision. A.03, Gaussian Inc., Wallingford, CT, **2016**.

[16] Grimme, S.; Ehrlich, S.; Goerigk, *L.  J. Comput. Chem.* **2011**, *33* (15), 1456–1465.

[17] A. V. Marenich, C. J. Cramer, D. G. Truhlar, *J. Phys. Chem. B* **2009**, *113*, 6378–6396.

[18] Lu, T. & Chen, F.  *J. Comput. Chem*., **2012**, *33*, 580-592.

[19] W. Humphrey, A. Dalke and K. Schulten, *J Mol Graph Model,* **1996**, *14*, 33-38.

[20] M. J. Frisch et al., Gaussian 16, Revision C.01, Gaussian, Inc., Wallingford CT, **2019**.

[21] T.Lu & F. Chen, *J. Comput. Chem.* **2012**, *33* (5), 580–592.

[22] C. I. Bayly et al., *J. Phys. Chem.* **1993**, *97* (40), 10269–10280.

[23] C. Wang et al., AuToFF Program, Version 1.0, Hzwtech, Shanghai, 2023.

[24] M. J. Abraham et al. *Software X* **2015**, 1–2, 19–25.

[25] H. J. C. Berendsen, D. Van Der Spoel & R. Van Drunen, *Comput. Phys. Commun.* **1995**, *91* (1–3), 43–56.

[26] B. Hess et al., *J. Comput. Chem.* **1997**, *18* (12), 1463–1472.

[27] W. Zhao et al., *J. Am. Chem. Soc.* **2009**, *131* (43).

[28] D. Van Der Spoel et al., *J. Phys. Chem. B* **2006**, *110* (9), 4393–4398.
